# Influence of blend proportion and baking conditions on the quality attributes of wheat, orange-fleshed sweet potato and pumpkin composite flour dough and bread: optimization of processing factors

**DOI:** 10.1007/s44187-023-00041-z

**Published:** 2023-02-16

**Authors:** Solomon Kofi Chikpah, Joseph Kudadam Korese, Oliver Hensel, Barbara Sturm, Elke Pawelzik

**Affiliations:** 1 Department of Agricultural and Biosystems Engineering, Faculty of Organic Agricultural Sciences, University of Kassel, Nordbahnhoftsrasse 1a, 37213 Witzenhausen, Germany; 2 Department of Food Science and Technology, Faculty of Agriculture, Food and Consumer Sciences, University for Development Studies, Nyankpala Campus, Post Office Box TL 1882, Tamale, Ghana; 3 Department of Agricultural Mechanization and Irrigation Technology, Faculty of Agriculture, Food and Consumer Sciences, University for Development Studies, Nyankpala Campus, Post Office Box TL 1882, Tamale, Ghana; 4 Leibniz Institute for Agricultural Engineering and Bioeconomy (ATB), Max-Eyth-Allee 100, 14469 Potsdam, Germany; 5 Albrecht Daniel Thaer-Institute of Agricultural and Horticultural Sciences, Humboldt-Universität Zu Berlin, Hinter der Reinhardtsstrasse 6–8, 10115 Berlin, Germany; 6 Division Quality of Plant Products, Department of Crop Science, Faculty of Agricultural Sciences, Georg-August-Universität Göttingen, Carl-Sprengel-Weg 1, 37075 Göttingen, Germany

**Keywords:** Dough development time, Baking conditions, Bread physical properties, Textural profile, Underutilized crops

## Abstract

Orange-fleshed sweet potato (OFSP) and pumpkin fruit are underutilized crops with great potential for the production of high-quality bread with health-enhancing properties. However, the incorporation of nonconventional flour in bread formula may influence the dough and bread quality properties. This study investigated the effect of partial substitution of wheat flour with OFSP (10–50%) and pumpkin flour (10–40%), baking temperature (150–200 °C) and baking time (15–25 min) on the quality properties of the composite dough and bread using response surface methodology (RSM). Dough rheological, bread physical and textural properties were analyzed, modelled and optimized using RSM. Satisfactory regression models were developed for the dough and bread quality attributes (R^2^ > 0.98). The dough development time, crust redness, hardness, and chewiness values increased while optimum water absorption of dough, specific volume, lightness, springiness, and resilience of bread decreased significantly (p < 0.05) with increasing incorporation of OFSP and pumpkin flour in the bread formula. Additionally, the specific volume, crust redness, crumb hardness, and chewiness of the composite bread increased significantly (p < 0.05) with increasing baking temperature from 150 to 180 °C but reduced at higher baking temperatures (≥ 190 °C). The staling rate declined with increased OFSP and pumpkin flour whereas increasing the baking temperature and time increased the bread staling rate. The optimized formula for the composite bread was 78.5% wheat flour, 11.5% OFSP flour, 10.0% pumpkin flour, and baking conditions of 160 °C for 20 min. The result of the study has potential applications in the bakery industry for the development of functional bread.

**Graphical Abstract:**

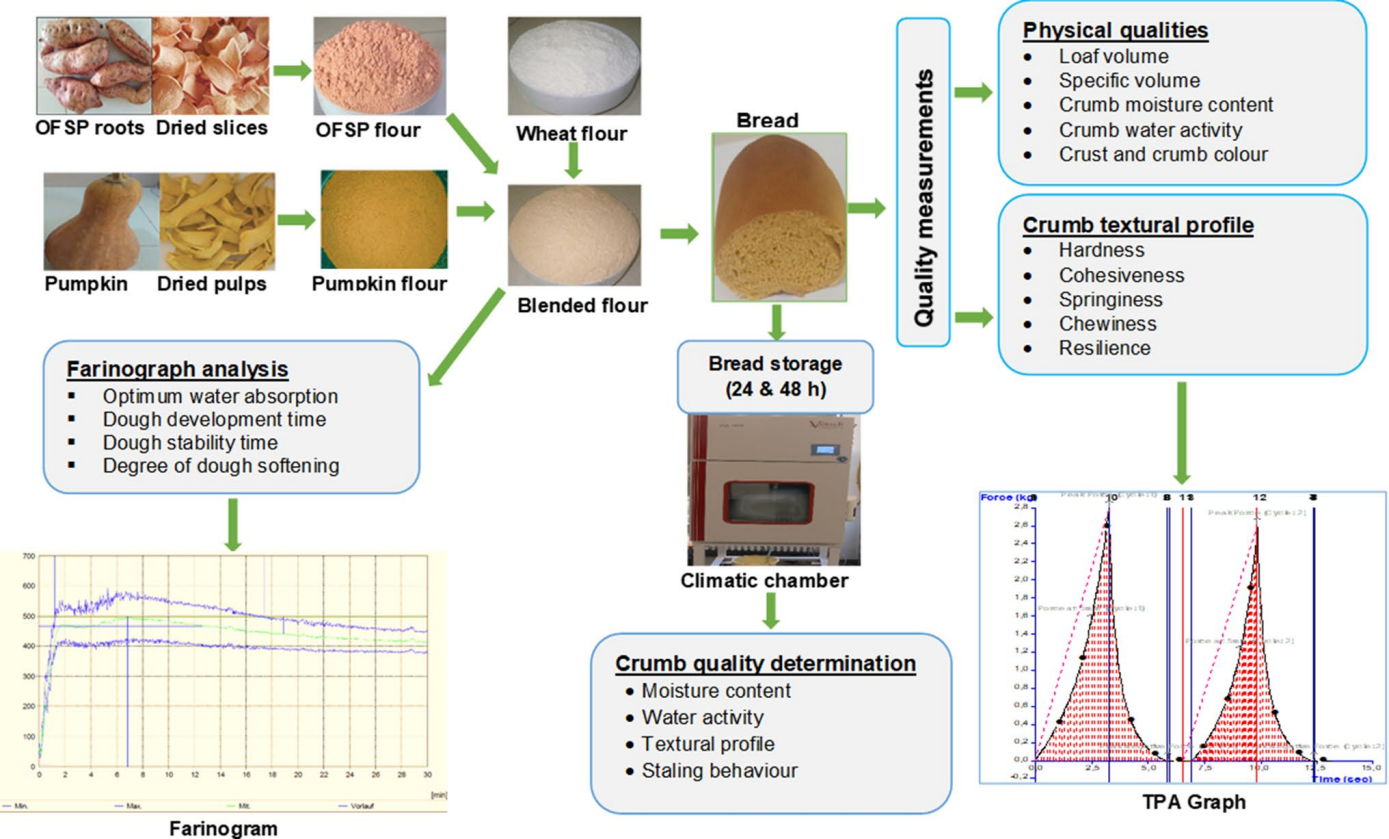

## Introduction

Adequate consumption of vital food nutrients and bioactive compounds such as vitamins, dietary fibre, carotenoids, polyphenols, flavonoids, and polysaccharides is crucial for good health [1–3]. The sufficient intake of natural bioactive compounds not only prevents type 2 diabetes, cancer, and cardiovascular diseases [3] but also strengthens the human immune system against viral infections such as the SARS-CoV-2 virus, the causal agent of the Covid-19 diseases [1, 4, 5]. However, the recent increase in global food crises due to the adverse impact of climate change, the Covid-19 pandemic and conflicts on the global economy, food production and supply is a threat to food security and health [6–8]. In 2021, about 702–824 million of the global population were affected by hunger and 2.3 billion people representing 29.3% of the world population were faced with moderate or severe food insecurity [6]. Therefore, there is a pressing need for policy reforms and innovative technologies in the food sector to increase food production and supply to meet current and future food demand [6, 7]. It was obvious that the increasing production of the major stable crops namely maize, wheat, rice and potato may not be sufficient to feed the growing population by the year 2030 due to the growing adverse impact of climate change, economic predicaments and increasing demand for these crops for livestock feeding [9, 10]. Previous studies have suggested that the diversification to include neglected and underutilized crops in the development of popularly consumed foods could greatly improve food and nutrition security, especially in sub-Saharan Africa [9, 11]. For example, orange-fleshed sweet potato (OFSP) is a rich source of ß-carotene, phenolics, flavonoids, vitamins, carbohydrates, and minerals [12, 13]. Similarly, pumpkin (*Cucurbita* spp) is rich in ß-carotene, dietary fibre, vitamin C, polyphenolics and flavonoid compounds [14]. Despite the nutritional value, these crops are underutilized in sub-Saharan Africa. Therefore, processing OFSP and pumpkin into quality flour for use in the development of highly consumed food products could help to improve food security [9, 11] while increasing the intake of bioactive compounds improve health and immune systems [1].

Bread is a highly consumed baked food among different groups globally as a major source of energy [15, 16]. However, wheat flour which is the key ingredient for bread making is imported into sub-Saharan Africa making the flour and its products very expensive [17, 18]. In recent years, consumer demand for safe, healthy and functional foods for enhancing good health and the immune system has increased tremendously since the outbreak of the Covid- 19 pandemic [7]. Nonetheless, wheat flour is low in health-promoting bioactive compounds [12, 19]. Therefore, the incorporation of OFSP and pumpkin flour in bread making would not only reduce the utilization of wheat flour and the cost of bread production in Sub-Saharan Africa [18] but also improve the nutritional and health-promoting benefits of the bread product [16, 20, 21]. However, the incorporation of nonconventional flour such as OFSP and pumpkin flour in the bread-making could influence the rheological properties of dough and the baking performance of bread [17, 18, 21–23]. According to Rosales-Juárez et al. [24], bread quality characteristics such as loaf-specific volume, bread colour, textural properties and flavour are largely dependent on wheat flour quality and bread-making processes. For example, the gluten in wheat flour is vital for the viscoelastic and carbon dioxide gas retention capacity of bread dough during fermentation and the initial baking stages [25, 26] and consequently determines the loaf volume [27] and textural properties of the bread crumb [28]. Previous studies have revealed that the baking process influences the quality attributes of freshly baked bread [29] and post-baking changes in the product’s quality parameters [17, 30]. The post-baking changes in bread such as moisture content loss or gain and modifications in texture could have unfavourable influences on consumer acceptability of the product [30]. Moreover, staling or firming of bread crumbs is a key factor contributing to economic losses for bread manufacturers and consumers [31]. Therefore, bread-making processes that minimize staling or losses of bread will be of great interest to both producers and consumers.

Several studies were performed on the development of new bread products using nonwheat flour ingredients like potato [32], sweet potato [18, 21, 33], cassava, sweet potato and sorghum mixed flour [34], kenaf leaves powder [35], rice, sorghum, and corn flours [36], rice bran [37, 38], fermented rice bran and pumpkin puree [39], sorghum sourdough and nabag pulp powder [40], whey protein concentrate and psyllium husk [41]. However, most of the investigations were focused on the effect of incorporating non-wheat flour on the dough and bread quality characteristics. There is limited information on the influence of both flour proportions and baking conditions on the quality properties of wheat-OFSP-pumpkin composite bread. The objectives of this study were to investigate the influence of partial substitution of wheat flour with OFSP and pumpkin flour on the composite dough properties, and determine the effect of the flour mixture and baking conditions on the physical properties, textural profile and staling behaviour of the composite bread. Additionally, the study aimed to optimize the flour mixture and baking conditions for wheat, OFSP and pumpkin composite bread formulation using response surface methodology.

## Materials and methods

### Raw materials

Orange-fleshed sweet potato (OFSP) roots (*Ipomoea batatas L.cv.CRI-Apomuden*) harvested 100 days after planting were purchased from a commercial farm located in Dambai, Krachi East Municipality of Oti region, Ghana. Mature and ripened pumpkin fruits (*Cucurbita moschata*) were purchased from the local market of the Tamale Metropolis in the Northern region of Ghana. Wheat flour (type 550), fresh yeast (Frischback-Hefe), margarine, salt, and sugar used for bread preparation were obtained from the Aldi-Nord supermarket in the city of Goettingen, Germany.

### OFSP flour preparation

The procedure used for OFSP flour production was described in a previous study [12]. Non-damaged OFSP roots were selected, washed with clean water, peeled with a stainless steel knife and sliced to 3 mm thickness using a Ritter E16 slicer (Ritterwerk GmbH, Gröbenzell, Germany). The slices were immersed in a 0.5% sodium metabisulfite solution for 5 min after which the pretreated slices were slices arranged on perforated trays in a single layer and dried at an air temperature of 60 °C for 4 h in an HT Mini” cabinet dryer (Innotech-Ingenieursgesellschaft mbH, Altdorf Germany). The dried samples were packaged into high-density polyethylene (HDPE) bags and transported to the University of Gottingen, Germany for further processing. The dried OFSP slices were milled to 250 μm flour particle sizes using a laboratory ultra centrifugal mill (Retsch ZM 100, RETSCH GmbH & Co. KG, Germany) operated at 14,000 rpm. The flour was cooled at room temperature (23 ± 1 °C) for 30 min, packed into HDPE and stored at 4 ± 1 °C for further use.

### Pumpkin flour preparation

The procedure for the production of the dried pumpkin product was described in a previous study [14]. The pumpkin fruit was washed with clean water and the rind was separated with a stainless steel knife. The fruit was then cut open and the fibrous strands and seeds were isolated from the pulp. The pulp was sliced to an average thickness of 3.2 ± 0.1 mm and 10.0 ± 2 mm in length. The pumpkin slices were pretreated in 0.3% sodium metabisulfite solution for 5 min after which water was drained. The treated pulps were spread on trays in a single layer and dried at 60 °C for 5 h in HT Mini” cabinet dryer (Innotech-Ingenieursgesellschaft mbH, Altdorf Germany). Milling of the dried pumpkin pulp slices and flour storage were carried out as described in “OFSP flour preparation” Section.

### Experimental design

The influence of partial substitution of wheat with OFSP and pumpkin flours and different baking conditions on the composite dough and bread quality properties was studied using response surface methodology. The I-optimal (combined) design of the Design-Expert software version 11 (Stat-Ease Inc., Minneapolis, United States) was used to generate 27 experimental design points which consist of 16 actual model points, 5 replication and lack-of-fit points each and additional central repeated points. The low and high constraints of flour mixture were wheat flour (40–80%), OFSP flour (10–50%) and pumpkin flour (10–40%) while the baking temperature and time were between 150 and 200 °C and 15–25 min, respectively. Bread from 100% wheat flour was used as the control. The investigated response variables were dough farinograph properties, bread characteristics such as loaf volume, specific volume, moisture content, water activity, CIELAB colour coordinates (L*, a* and b*), and crumb textural profile.

### Bread preparation and storage

The experimental bread samples were prepared using the straight-dough baking procedure described in ICC standard method No.131 [42] with few alterations based on Kieffer et al. [43]. The bread-making formula based on 100 g flour (14% moisture basis) consists of fresh baker’s yeast (5.0%), sugar (1.0%), margarine (1.0%), salt (1.5%), and water (based on the farinograph optimum water absorption). The dough was prepared using a farinograph machine (Farinograph-E, Brabender, GmbH & Co. KG, Duisburg, Germany). The kneading time for dough preparation was based on the farinograph dough development time measured for each formulation. The dough was fermented in a proofer (UNOX XLT 133, Cadoneghe, Italy) for 20 min at 30 ± 1 °C and 85% relative humidity (RH). The fermented dough was divided into pieces (50 g), rounded by hand and allowed to rest at room temperature (23 ± 1 °C) for 3 min. The relaxed dough was rolled, moulded into a croissant-like shape, and proofed for 30 min at 30 ± 1 °C and 85% RH. A professional oven (Unox XFT133 ARIANNA, Cadoneghe, Italy) was used for the baking following the baking conditions for each formulation (Table 2). During the first minute of baking, steam (100 ml water) was injected into the oven. The bread quality characteristics were determined after cooling the freshly baked bread at room temperature for 2 h. Also, bread samples were packed in HDPE bags and stored for 24 h in a climatic chamber (VCL 4010, Vötsch Industrietechnik GmbH, Germany) set at a temperature of 25 °C and 50% RH.

### Proximate analysis of wheat, OFSP and pumpkin flour

The moisture, crude protein, crude fat, crude fibre, and ash contents of the flour was analyzed per the standard protocols of the Association of Official Analytical Chemists [44]. Each analysis was conducted in duplicate.

The total available carbohydrate was determined by the difference method shown below:

1 $${\text{Carbohydrate}} = 100 - \left( {{\text{\% moisture}} + {\text{\% protein}} + {\text{\% fat}} + {\text{\% fibre}} + {\text{\% ash}}} \right)$$

### Analysis of dough rheological characteristics

The farinograph test was performed on the wheat, OFSP, and pumpkin flour blends and the 100% wheat flour using a farinograph (Farinograph-E, Brabender, GmbH & Co. KG, Duisburg, Germany) following the American Association of Cereal Chemists Method 54–21 [45]. The farinograph parameters measured were optimum water absorption (%, the quantity of water needed to centre the farinograph curve on the 500- Brabender units, BU); dough development time (min, the mixing time needed to achieve maximum consistency); dough stability (min, the time the dough maintains optimal consistency), and dough degree of softening (BU, the decrease in dough optimum consistency after 12 min). Three replicated measurements were performed.

### Measurement of loaf volume and specific volume

The mass (g) of each loaf was measured 2 h after baking using a precision balance (KERN 572, KERN & SOHN GmbH, Germany) that has an accuracy of ( ±) 0.001 g. The loaf volume (cm^3^) was measured according to the rapeseed displacement procedure the standard method 10–05.01 of AACC [45]. The specific volume was computed using Eq. (2). All measurements were replicated three times.

2 $${\text{Specific volume}}\left( {{\raise0.7ex\hbox{${{\text{cm}}^{3} }$} \!\mathord{\left/ {\vphantom {{{\text{cm}}^{3} } {\text{g}}}}\right.\kern-0pt} \!\lower0.7ex\hbox{${\text{g}}$}}} \right) = { }\frac{{\text{loaf volume}}}{{\text{loaf mass}}}$$

### Flour and bread colour measurement

The CIELAB colour coordinates for lightness (L*), redness/greenness (a*) and yellowness/blueness (b*) of the raw flour and bread samples were measured with a chroma meter (CR400 Konica Minolta, Marunouchi, Japan) following the procedure described by Chikpah et al. [17]. The calibration of the equipment was done using a standard white plate at D65 illumination (Y = 80.1, x = 0.3219, and y = 0.3394). The crust and crumb colour values of the same bread sample were measured at five different points after the edges (3 mm) of the bread were taken off. The crust of the bread was completely isolated before the colour parameters of the crumb were determined. Five replicated colour measurements were taken.

### Analysis of crumb moisture content and water activity

The moisture content of bread crumbs was analyzed using the AACC air oven method 44–15.02, the one-stage procedure [46] with an electric oven (Memmert GmbH, Germany). The analysis of water activity was performed at room temperature (24 ± 1 °C) using a water activity meter (Labswift-aw, Novasina AG, Switzerland). Three replicated measurements were made.

### Analysis of crumb textural characteristics

Textural profile analysis (TPA) of the freshly baked bread and 24 h stored bread samples were measured with a texture analyzer (TA.XT plus, Stable Micro Systems Ltd, Godalming, UK) following the procedure described previously with slight modification [47]. The textural analyzer was connected to a desktop computer that was equipped with Texture Exponent 32 software (SMS Ltd) for test settings and data processing. The entire crust and about 3 mm of the loaf edges were taken off using a sharp stainless steel knife. The bread crumb was cut manually into slices having dimensions of 26 mm × 26 mm × 20 mm for breadth, length and height, respectively. A two-consecutive unidirectional compression test was performed on each slice using a 50 kg load cell and a 25-mm-diameter cylinder aluminium probe (SMS/P25). The TPA test settings applied were a trigger force of 0.098 N, a strain of 50%, a holding time of 1 s between compression cycles, and pretest, test and post-test speeds of 1, 2 and 2 mm/s, respectively. The crumb hardness (kg), cohesiveness (dimensionless), springiness (dimensionless), chewiness (kg), and resilience (dimensionless) were textural parameters measured from the force–time graph of the TPA. The crumb staling rate after 24 h of storage was calculated following Eq. (3). The textural analysis of each sample was replicated three times.

3 $${\text{Staling rate}} = \frac{{{\text{H}}_{2} - {\text{H}}_{1} }}{{{\text{H}}_{1} }}$$

where H_1_ and H_2_ represent the crumb hardness (kg) after 2 h of baking and 24 h of storage respectively*.*

### Optimization of processing factors

The numerical optimization of the flour mixture, baking temperature and time for wheat-OFSP-pumpkin composite bread formulation was carried out following the modified desirability function approach [48] using Design-Expert software version 11.1.2.0 (Stat-Ease Inc., Minneapolis, United States). This desirability function approach which uses mathematical methods to transform a multi-response optimization problem into a univariate problem is suitable for the simultaneous optimization of numerous response variables [48–51]. This optimization method transforms each response variable (*Y*_*i*_) to a desirability value (*d*_*i*_); where values of *d*_*i*_ range from 0 (unfavourable) to 1 (most favourable). A global desirability value was then computed using Eq. (4).

4 $${\text{D}} = \left[ {{\text{d}}_{1} \times {\text{d}}_{2} \times {\text{d}}_{3} \ldots ..{\text{d}}_{{\text{k}}} } \right]^{{{\raise0.7ex\hbox{$1$} \!\mathord{\left/ {\vphantom {1 {\text{k}}}}\right.\kern-0pt} \!\lower0.7ex\hbox{${\text{k}}$}}}}$$

where D is the global desirability, d_1_, d_2_, d_3_….d_k_ represents the individual desirability value and k = the number of response variables. Similarly, the values of D ranged from 0 (unacceptable product) to 1 (most acceptable product). Therefore, to formulate an acceptable product the values of d_i_ must be greater than zero [48].

### Statistical analysis

The experimental data were analyzed using the response surface methodology of the Design-Expert software version 11.1.2.0 (Stat-Ease Inc., Minneapolis, United States) to determine the influence of the processing factors (flour proportion and baking conditions) on the investigated dough and bread quality attributes. The combined model fit summary and the model fitness statistics of the analysis of variance (ANOVA) were used in the selection of suitable models to describe the relationship between the processing factors and response variables. The best-fitted model was selected based on a model with the highest significant terms (p < 0.05), highest coefficient of determination (*R*^*2*^), adjusted R^2^, prediction R^2^ and adequate precision values and insignificant lack-of-fit (p > 0.05) [17, 52]. The normality of the data was verified using the normal residuals plot, Cook’s distance, and Box-Cox plot. In some cases, the backward elimination regression with the p-value criterion (alpha = 0.1) was used to refine models that have numerous insignificant terms. Besides, One-way analysis of variance (ANOVA) and Tukey’s pairwise comparison were conducted to determine the significant difference (p < 0.05) between the quality attributes of dough and bread of the various formulations. Furthermore, Principal Component Analysis (PCA) was performed to discriminate between the different bread formulations and also to identify the association between the dough and bread quality characteristics. The ANOVA and PCA were performed in an SPSS software (IBM SPSS Statistics, version 25) and XLSTAT (Version 2018.1, Addinsoft, 2018) respectively.

## Results and discussion

### Physicochemical characteristics of the flour

The physicochemical properties of flour influence the dough properties and quality attributes of the final baked bread [23]. The proximate composition and CIELAB L*, a*, b* colour parameters of wheat, OFSP and pumpkin flour utilized in this study are presented in Table 1*.* Similar to the findings by Chikpah et al. [12], wheat flour had the highest values for crude protein and moisture content and the lowest crude fibre, total ash, and carbohydrate contents as compared with the OFSP and pumpkin flour. The pumpkin flour had the highest fat, crude fibre and total ash values while OFSP flour obtained the largest value for carbohydrates. In terms of colour, the highest value for L* (91.20 ± 0.17) was observed in the wheat flour while the pumpkin flour had the least value L* value (63.80 ± 0.54). The OFSP flour was more reddish while the pumpkin flour was more yellowish as showed by their a* and b* values respectively (Table 1). The colour values measured for wheat, OFSP and pumpkin flours were similar to the values reported in previous studies [12, 14]. The higher a* and b* values measured in the OFSP and pumpkin flour could be attributed to the natural pigments such as carotenoids in OFSP [9–13] and pumpkin fruit [14].

**Table 1:** Proximate composition^1^ and CIELAB colour^2^ of wheat, OFSP and pumpkin flour

Parameter	Wheat flour	OFSP flour	Pumpkin flour
Moisture content (%)	12.74 ± 0.15^c^	7.23 ± 0.07^a^	8.10 ± 0.10^b^
Crude protein (%)	11.95 ± 0.08^c^	5.16 ± 0.02^a^	5.92 ± 0.05^b^
Crude fibre (%)	0.39 ± 0.02^a^	3.68 ± 0.04^b^	4.35 ± 0.08^c^
Fat (%)	1.03 ± 0.01^b^	0.59 ± 0.01^a^	1.47 ± 0.02^c^
Total ash (%)	0.87 ± 0.01^a^	1.27 ± 0.09^b^	2.96 ± 0.07^c^
Carbohydrate (%)	73.02 ± 0.17^a^	82.07 ± 0.22^c^	77.20 ± 0.13^b^
L*	91.20 ± 1.26^c^	69.27 ± 0.41^b^	63.80 ± 0.54^a^
a*	−0.08 ± 0.01^a^	18.93 ± 0.18^c^	9.36 ± 0.12^b^
b*	11.86 ± 0.24^a^	35.70 ± 0.29^b^	49.82 ± 0.63^c^

Values in a row with no common superscript are significantly different (p < 0.05). L* represent lightness (L* = 100 is white and 0 is dark)

^1^Value is presented as an average ± standard deviation (n = 3) and expressed on a dry matter basis

except for moisture

^2^Value is an average ± standard deviation (n = 5)

a*is redness (a > 0) or greenness (a < 0) and b* is yellowness (b* > 0) or blueness (b* < 0)

### Dough rheological properties

**Fig. 1:**
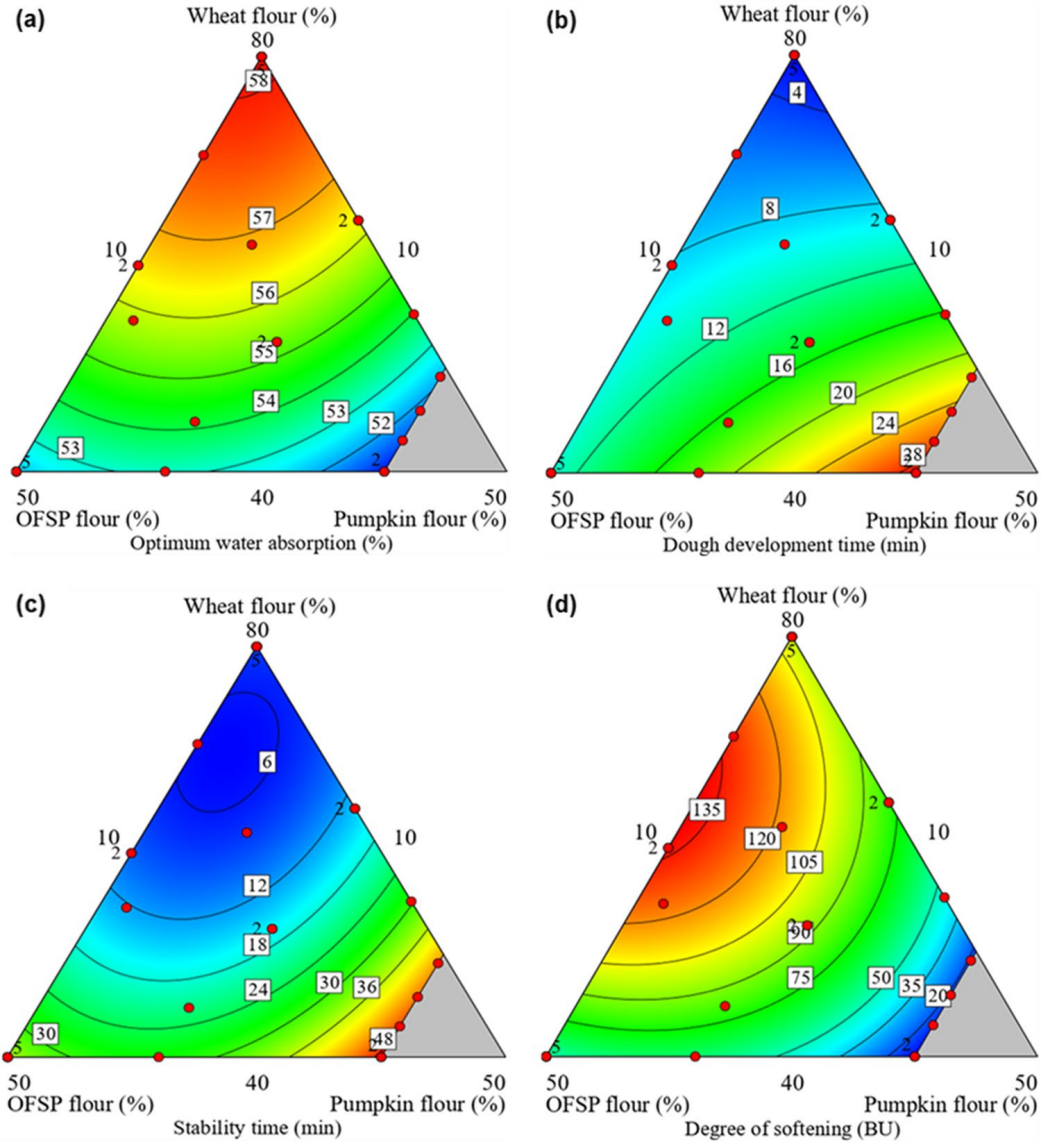
Contour plots showing the influence of wheat, OFSP and pumpkin flour proportions on farinograph optimum water absorption (**a**), dough development time (**b**), stability time (**c**), degree of softening (**d**) of the composite bread dough. The red round marks represent the design points

The rheological analysis of dough is crucial because it reveals the dough mixing or handling properties of dough and is also useful for predicting the baking performance [53]. The flour proportions greatly affected (p < 0.05) the farinograph properties of the composite dough (Table 2). The optimum water absorption (OWA) and dough development time (DDT) of the OFSP and pumpkin composite flour doughs ranged from 50.8 to 58.2%, and 2.6 to 29.2 min, respectively. The control dough (100% wheat flour) had a significantly higher value for OWA (60.1 ± 0.2%) but a shorter DDT value (2.1 ± 0.1 min) as compared with the composite flour doughs. While OWA is the quantity of water required for consistent dough formation, DDT shows the mixing time required for consistent dough formation and further describes the rate of formation of gluten-network during dough mixing [47]. In this study, the OWA increased while DDT increased with the partial substitution of wheat flour with OFSP and pumpkin (Fig. 1a and b). Similar observations were reported by Kundu et al. [54] and Bultum et al. [38] when wheat flour was partially replaced with pumpkin flour and rice bran respectively. Moreover, the substitution of wheat flour with wheat bran resulted in increased DDT [47]. The decline in OWA of composite flour dough could be associated with the dilution of gluten as a result of the partial substitution of wheat flour with non-gluten flour [26]. Also, a decrease in gluten and an increase in fibre-water interaction can slow the rate of gluten network formation and subsequently prolong DDT [55]. The dough stability time (ST) and degree of softening (DOS) of the composite flour dough varied from 5.1 to 50.0 min for ST, and 9.0–138.0 BU for DOS (Table 2). The farinograph dough stability and degree of softening indicate the strength and resistance of dough against mixing. Similar to the previous findings by Turksoy & Özkaya [56] and Kundu et al. [54], the values for ST declined while DOS increased as the proportions of OFSP and pumpkin flour increased (Fig. 1c and d). The prolonged stability of the composite dough can be attributed to the interaction between water and flour components like starch and fibre which creates a more stable and resistant dough to the mixing force [54].

**Table 2:** Rheological properties of doughs from wheat, OFSP and pumpkin blended flours

Experimentalrun	Wheat flour (%)	OFSP flour (%)	Pumpkin flour (%)	OWA (%)	DDT (min)	ST (min)	DOS (BU)
F1	52.5	22.5	25.0	55.3 ± 0.2^g^	11.8 ± 0.1^h^	13.9 ± 0.4^i^	92.8 ± 0.5^f^
F2	40.0	50.0	10.0	52.2 ± 0.1^j^	10.5 ± 0.3^i^	34.8 ± 0.3^e^	52.0 ± 0.3^k^
F3	61.9	19.8	18.3	56.9 ± 0.1^d^	9.2 ± 0.2^k^	8.6 ± 0.1^l^	119.5 ± 0.7^d^
F4	54.6	33.2	12.2	55.8 ± 0.1^f^	9.9 ± 0.1^j^	12.5 ± 0.1^j^	122.0 ± 0.4^c^
F5	80.0	10.0	10.0	58.1 ± 0.1^b^	2.7 ± 0.1^n^	7.8 ± 0.1^m^	97.5 ± 0.7^e^
F6	40.0	20.0	40.0	50.8 ± 0.1^m^	29.0 ± 0.1^a^	49.7 ± 0.2^a^	9.0 ± 0.2^o^
F7	55.2	10.0	34.8	54.0 ± 0.2^h^	16.1 ± 0.2^g^	29.8 ± 0.3^f^	44.8 ± 0.8^l^
F8	40.0	50.0	10.0	52.1 ± 0.3^j^	10.8 ± 0.1^i^	34.6 ± 0.1^e^	51.9 ± 0.2^k^
F9	40.0	37.9	22.1	53.2 ± 0.1^i^	18.1 ± 0.2^e^	27.8 ± 0.4^g^	60.4 ± 0.4^j^
F10	80.0	10.0	10.0	58.2 ± 0.2^b^	2.8 ± 0.1^n^	7.9 ± 0.1^m^	97.6 ± 0.7^e^
F11	80.0	10.0	10.0	58.0 ± 0.1^b^	2.6 ± 0.3^n^	7.8 ± 0.1^m^	97.2 ± 0.3^e^
F12	40.0	20.0	40.0	51.0 ± 0.1^m^	29.2 ± 0.3^a^	50.0 ± 0.3^a^	9.7 ± 0.4^o^
F13	40.0	50.0	10.0	52.0 ± 0.1^jk^	10.7 ± 0.1^i^	35.0 ± 0.4^e^	52.1 ± 0.1^k^
F14	43.0	17.0	40.0	51.5 ± 0.1^l^	26.6 ± 0.1^b^	46.2 ± 0.3^b^	15.9 ± 0.2^n^
F15	64.2	10.0	25.8	56.3 ± 0.1^e^	9.2 ± 0.1^k^	13.4 ± 0.1^i^	78.8 ± 0.3^h^
F16	52.5	22.5	25.0	55.1 ± 0.3^g^	14.6 ± 0.1^h^	13.8 ± 0.1^i^	92.5 ± 0.6^f^
F17	44.8	33.0	22.2	54.2 ± 0.1^h^	16.8 ± 0.1^f^	21.7 ± 0.1^h^	82.1 ± 0.2^g^
F18	40.0	50.0	10.0	52.2 ± 0.1^j^	10.7 ± 0.2^i^	34.8 ± 0.2^e^	51.3 ± 0.3^k^
F19	59.9	30.1	10.0	56.5 ± 0.3^e^	8.3 ± 0.1^l^	9.5 ± 0.1^l^	138.0 ± 0.7^a^
F20	80.0	10.0	10.0	58.0 ± 0.1^b^	2.9 ± 0.1^n^	7.7 ± 0.1^m^	98.2 ± 0.3^e^
F21	40.0	50.0	10.0	52.3 ± 0.2^j^	10.7 ± 0.1^i^	35.1 ± 0.1^e^	51.7 ± 0.5^k^
F22	70.5	19.5	10.0	57.7 ± 0.3^c^	5.9 ± 0.2^m^	6.1 ± 0.1^n^	131.0 ± 0.8^b^
F23	59.9	30.1	10.0	56.4 ± 0.2^e^	8.4 ± 0.1^l^	9.6 ± 0.1^k^	137.2 ± 0.3^a^
F24	45.9	14.1	40.0	51.8 ± 0.1^k^	24.6 ± 0.1^c^	44.5 ± 0.3^c^	18.3 ± 0.2^m^
F25	64.2	10.0	25.8	56.2 ± 0.1^e^	9.2 ± 0.1^k^	13.8 ± 0.2^i^	79.4 ± 0.3^h^
F26	80.0	10.0	10.0	58.1 ± 0.3^b^	2.6 ± 0.1^n^	7.9 ± 0.1^m^	97.9 ± 0.5^e^
F27	49.2	10.8	40.0	52.2 ± 0.1^j^	22.2 ± 0.3^d^	42.7 ± 0.2^d^	19.1 ± 0.4^m^
Control	100.0	0.0	0.0	60.3 ± 0.2^a^	2.1 ± 0.1^o^	10.1 ± 0.4^k^	72.5 ± 0.6^i^

Model	Quadratic	Quadratic	Quadratic	Quadratic
F-value (model)	3916.09^***^	12730.63^***^	11526.19^***^	8241.30^***^
F-value (lack-of-fit)	0.573^ ns^	1.440^ ns^	1.940^ ns^	1.586^ ns^
Coefficient of determination (R^2)^	0.9935	0.9968	0.9927	0.9943
Adjusted-R^2^	0.9870	0.9915	0.9881	0.9896

Values for the dough quality parameters are presented as average ± standard deviation computed from three replicated measurements (n = 3). Values within the same column that have different letters are significantly different (p < 0.05)

*OWA* optimum water absorption, *DDT* dough development time, *ST* stability time, *DOS* degree of softening

^***^P < 0.0001; ns means not significant (p > 0.05)

The farinograph dough parameters were adequately described by the quadratic model. The model fitness statistics indicated the model and the terms (A, B, C, AB, AC and BC) were significant (P < 0.0001), the values of R^2^ and adjusted R^2^ are greater than 0.98 and the lack-of-fit was insignificant (p > 0.05) as indicated Table 2. The quadratic model for describing the dough OWA and DDT values as influenced by the proportions of wheat flour (A), OFSP flour (B) and pumpkin flour (C) in terms of actual settings are shown in Eqs. (5) and (6), respectively.

5 $${\text{OWA }}\left( {\text{\% }} \right) = 0.5719{\text{A}} + 0.3097{\text{B}} + 0.1240{\text{C}} + 0.0038{\text{AB}} + 0.0057{\text{AC}} + 0.0065{\text{BC}}$$

6 $${\text{DDT }}\left( {{\text{min}}} \right) = 0.0053{\text{A}} - 0.0855{\text{B}} + 1.3187{\text{C}} + 0.0039{\text{AB}} - 0.0164{\text{AC}} + 0.0013{\text{BC}}$$

### Physical properties of bread

**Fig. 2:**
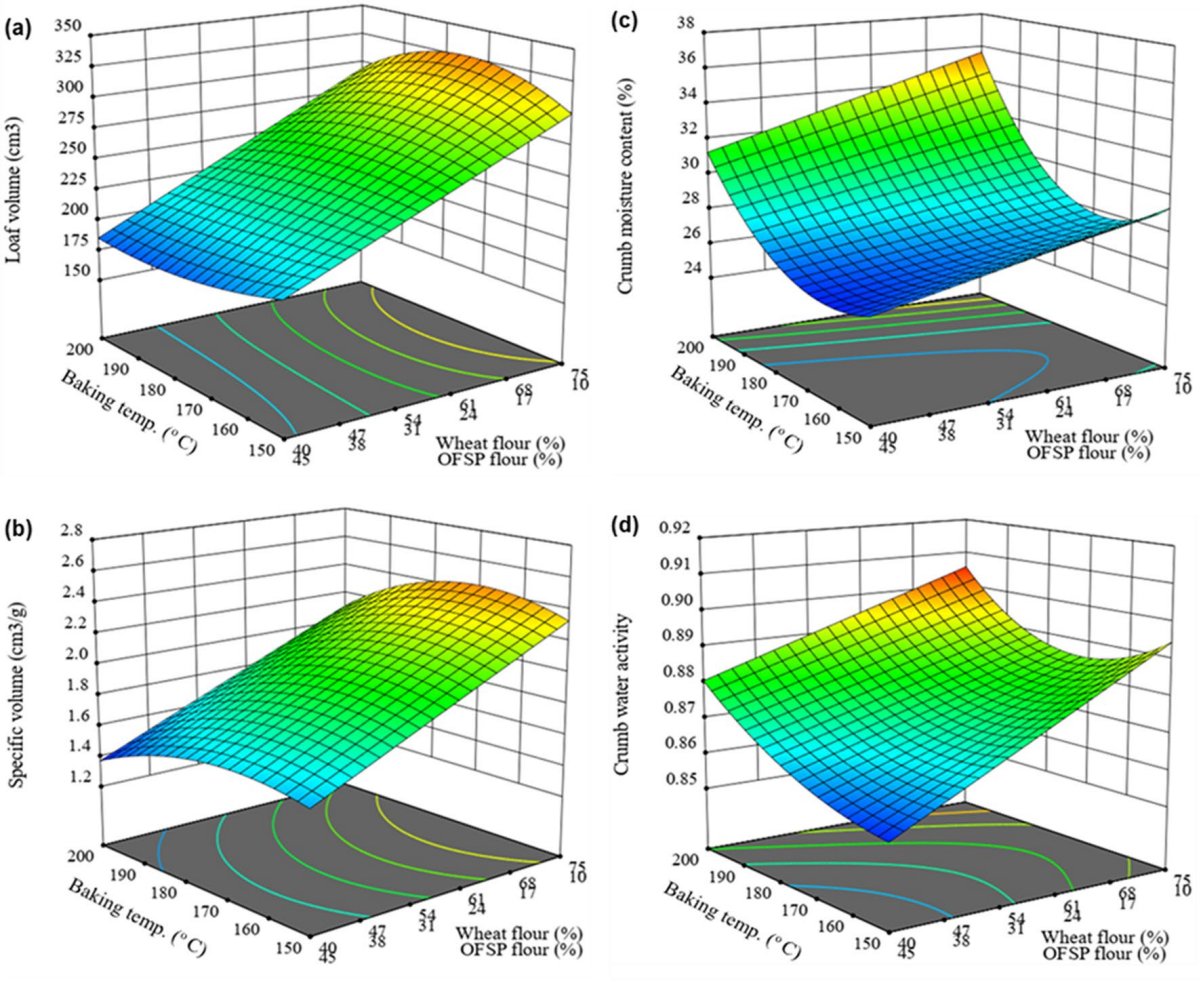
Response surface plots showing the effects of wheat OFSP flour proportions, pumpkin flour (15%), baking temperature and baking time (19 min) on loaf volume (**a**), specific volume (**b**), crumb moisture content (**c**), and water activity (**d**) of the composite bread

Table 3 presents the physical properties of the control and composite bread samples. The loaf volume is vital quality property of bread and indicates the gas hold capacity of bread dough [47]. In the present study, the control bread had a significantly (p < 0.001) higher loaf volume (363.78 ± 1.19 cm^3^) and specific volume (2.86 ± 0.02 cm^3^/g) as compared with the loaf volume of 166.50–349.94 cm^3^ and specific volume of 1.34–2.62 cm^3^/g for the composite bread products. Similar to the findings made in wheat-potato steamed bread [57], wheat-OFSP composite bread [18], and pumpkin-wheat composite [22], the loaf volume and specific volume of the experimental bread samples decreased with increasing substitution of wheat flour with OFSP and pumpkin flour as shown in Fig. 2. This phenomenon can be ascribed to the dilution of gluten-forming proteins due to the partial substitution of wheat flour with OFSP and pumpkin flour since the formation of a gluten network is crucial for the viscoelastic property and gas-holding ability of dough [25–27]. Furthermore, the high fibre content of the OFSP flour and pumpkin flour could disrupt gluten network formation and collapse gas-holding cells and hence reduce loaf volume [28, 47].

**Table 3:** Physical characteristics of wheat-OFSP-Pumpkin composite bread and the ANOVA results for the mixture × process model

Experimental runs	Wheat flour (%)	OFSP flour (%)	Pumpkin flour (%)	Baking temperature (^o^ C)	Baking time (min)	Loaf volume (cm^3^/100 g flour)	Specific volume (cm^3^/g)	Crumb moisture content (%)	Crumb a_w_
F1	52.5	22.5	25.0	170	19	232.59 ± 1.29^n^	1.87 ± 0.03^j^	27.27 ± 0.04^pq^	0.867 ± 0.002^ijk^
F2	40.0	50.0	10.0	200	17	177.60 ± 1.02^w^	1.44 ± 0.01^q^	29.15 ± 0.03^m^	0.872 ± 0.001^i^
F3	61.9	19.8	18.3	190	23	247.51 ± 1.41^l^	1.94 ± 0.02^i^	30.34 ± 0.01^j^	0.890 ± 0.001^fg^
F4	54.6	33.2	12.2	150	19	246.70 ± 1.07^m^	1.86 ± 0.01^j^	27.25 ± 0.05^pq^	0.871 ± 0.001^ij^
F5	80.0	10.0	10.0	180	17	349.94 ± 1.13^b^	2.60 ± 0.01^b^	30.71 ± 0.06^i^	0.894 ± 0.001^ef^
F6	40.0	20.0	40.0	150	17	169.60 ± 1.55^y^	1.49 ± 0.03^p^	25.20 ± 0.03^w^	0.860 ± 0.001^lm^
F7	55.2	10.0	34.8	180	23	200.73 ± 0.98^p^	1.74 ± 0.03^k^	28.29 ± 0.02^n^	0.878 ± 0.003^h^
F8	40.0	50.0	10.0	150	25	204.42 ± 1.06^o^	1.58 ± 0.03^m^	25.49 ± 0.05^v^	0.854 ± 0.001^n^
F9	40.0	37.9	22.1	200	15	191.00 ± 1.27^q^	1.52 ± 0.01^o^	36.85 ± 0.02^b^	0.892 ± 0.001^efg^
F10	80.0	10.0	10.0	200	15	310.80 ± 1.64^e^	2.38 ± 0.02^d^	37.33 ± 0.04^a^	0.908 ± 0.001^b^
F11	80.0	10.0	10.0	200	23	297.62 ± 1.17^g^	2.30 ± 0.04^e^	34.59 ± 0.02^e^	0.901 ± 0.001^cd^
F12	40.0	20.0	40.0	180	21	166.50 ± 1.48^z^	1.36 ± 0.03^r^	29.64 ± 0.03^l^	0.866 ± 0.001^jk^
F13	40.0	50.0	10.0	190	17	179.30 ± 1.14^u^	1.55 ± 0.02^n^	27.47 ± 0.01^o^	0.869 ± 0.001^ijk^
F14	43.0	17.0	40.0	170	21	178.43 ± 1.00^v^	1.59 ± 0.02^m^	27.11 ± 0.02^q^	0.857 ± 0.001^mn^
F15	64.2	10.0	25.8	160	21	270.80 ± 1.31^h^	2.23 ± 0.03^f^	26.70 ± 0.03^r^	0.879 ± 0.001^h^
F16	52.5	22.5	25.0	170	19	232.54 ± 1.29^n^	1.86 ± 0.03^j^	27.33 ± 0.01^op^	0.866 ± 0.002^jk^
F17	44.8	33.0	22.2	200	19	180.68 ± 1.42^t^	1.43 ± 0.01^q^	35.27 ± 0.01^d^	0.897 ± 0.001^de^
F18	40.0	50.0	10.0	150	15	187.51 ± 0.87^r^	1.54 ± 0.02^no^	26.50 ± 0.04^s^	0.864 ± 0.001^kl^
F19	59.9	30.1	10.0	160	15	268.80 ± 1.34^i^	2.13 ± 0.01^h^	30.01 ± 0.03^k^	0.881 ± 0.001^h^
F20	80.0	10.0	10.0	170	21	340.47 ± 1.09^c^	2.62 ± 0.03^b^	26.74 ± 0.03^r^	0.889 ± 0.001^fg^
F21	40.0	50.0	10.0	200	25	171.50 ± 1.16^x^	1.34 ± 0.03^r^	28.31 ± 0.01^n^	0.872 ± 0.001^i^
F22	70.5	19.5	10.0	190	15	299.72 ± 0.85^f^	2.31 ± 0.01^e^	33.16 ± 0.02^g^	0.888 ± 0.003^g^
F23	59.9	30.1	10.0	160	25	262.01 ± 1.51^k^	2.20 ± 0.02^g^	26.00 ± 0.02^t^	0.878 ± 0.001^h^
F24	45.9	14.1	40.0	160	17	184.30 ± 1.12^s^	1.59 ± 0.02^m^	25.82 ± 0.02^u^	0.853 ± 0.001^n^
F25	64.2	10.0	25.8	150	21	265.41 ± 1.37^j^	2.25 ± 0.02^f^	28.40 ± 0.02^n^	0.881 ± 0.001^h^
F26	80.0	10.0	10.0	150	15	328.30 ± 1.25^d^	2.48 ± 0.03^c^	36.51 ± 0.02^c^	0.905 ± 0.001^bc^
F27	49.2	10.8	40.0	180	15	190.72 ± 1.08^q^	1.71 ± 0.01^l^	33.46 ± 0.03^f^	0.872 ± 0.001^i^
Control	100.0	0.0	0.0	200	21	363.78 ± 1.19^a^	2.86 ± 0.02^a^	31.20 ± 0.04^h^	0.916 ± 0.003^a^

Cross model (flour mixture × baking conditions)	Linear × Quadr	Linear × Quadr	Linear × Quadr	Reduced linear × Quadr
Model (F-value)	133.91^***^	1354.14^***^	1194.57^***^	97.38^***^
Lack of fit (F-value)	93.880^ ns^	0.331^ ns^	25.370^ ns^	10.365^ ns^
Coefficient of determination (R^2)^	0.9961	0.9975	0.9906	0.9784
Adjusted R^2^	0.9886	0.9939	0.9890	0.9758

Values indicate average ± standard deviation computed from three replicated measurements (n = 3). Values within the same column that have different letters are significantly different (p < 0.05)

^***^ P < 0.0001, ns means not significant (p > 0.05) and Quadr Quadratic

Moisture content and water activity (a_w_) are vital quality attributes of bread due to the effect on the microbial activity, textural and sensory properties of bread [23]. The moisture content of the crumb for the control bread was 31.20 ± 0.04%, and 25.20–37.33% for the composite bread. The composite bread crumbs had a significantly (p < 0.05) lower a_w_ (0.853–0.908) than the control bread (0.916 ± 0.003) as presented in Table 3. Similar to the result reported for wheat flour and OFSP puree composite bread [23], a decrease in a_w_ was observed in the wheat, OFSP and pumpkin flour composite bread as the proportions of the nonwheat flour increased. This can be attributed to fibre and sugar in OFSP and pumpkin flour since these chemical components can bind to water and reduce the availability of free water in the food material [17, 20].

The physical properties of the composite bread were greatly affected by the baking temperature and time. The quality properties of bread are dependent on the wheat flour quality [58], heating intensity and duration of baking [29, 59]. Similar to the findings of other studies [29, 60], loaf volume and specific volume increase as the baking temperature rises from 150 to 180 °C (Fig. 2a and b). This phenomenon could be attributed to an increase in CO_2_ gas expansion in the dough due to an increase in the heating rate at the early stage of baking [29, 61]. Nonetheless, at higher baking temperatures (≥ 180 °C) the loaf volume and specific volume declined significantly. This can be ascribed to an early occurrence of starch gelatinization at higher baking temperatures which have the potential of reducing dough extensibility and causing rupture of gas cell membranes and finally decreasing the volume of the baked bread [61, 62]. The crumb moisture content and a_w_ were reduced slightly with an increase in baking temperature up to 180 °C after which the moisture retention and a_w_ increased with increasing baking temperature (Fig. 2c and d). Since an early starch gelatinization and hardening of the bread crust may occur at higher baking temperatures this has the potential to reduce moisture movement from the bread crumb to the surface due to low water diffusivity through a gelatinized starch medium [63]. A continuous decreasing trend in crumb moisture content and a_w_ was observed with a prolonged baking time. This result was consistent with a previous study [29].

A linear × quadratic cross-model for flour mixture and baking conditions respectively was the most adequate model for defining the physical quality attributes of the composite bread as influenced by flour proportions and baking conditions. The model statistics are shown in Table 3 whereas the model equations for describing specific volume (SV) and crumb moisture content (MC) are indicated in Eqs. (7) and (8), respectively.

7 $$\begin{aligned} {\text{SV~}}\left( {{\text{cm}}^{3} /{\text{g}}} \right) = & - 0.07512{\text{A}} - 0.043674{\text{B}} - 0.141352{\text{C}} + 0.001739{\text{AD}} + 0.004674{\text{AE}} \\ & + 0.000618{\text{BD}} - 0.000172{\text{BE}} - 0.000526{\text{CD}} + 0.021697{\text{CE}} + 0.00000775{\text{ADE}} \\ & + {\text{~}}0.000008{\text{BDE}} - 0.000104{\text{CDE}} - 0.00000545{\text{AD}}^{2} + 0.000088{\text{AE}}^{2} - {\text{~~}}0.0000022{\text{BD}}^{2} \\ & - 0.000036{\text{BE}}^{2} + 0.00000658{\text{CD}}^{2} - 0.000095{\text{CE}}^{2} \\ \end{aligned}$$

8 $$\begin{aligned} {\text{MC~}}\left( {\text{\% }} \right)\, = \, & 4.015857{\text{A}} - 1.267481{\text{B}} - 0.243906{\text{C}} - 0.024166{\text{AD}} - 0.160662{\text{AE}} \\ & + 0.011665{\text{BD}} + 0.049109{\text{BE}} - 0.042043{\text{CD}} + 0.368722{\text{CE}} + 0.000391{\text{ADE}} \\ & - 0.000031{\text{BDE}} - 0.001433{\text{CDE}} + 0.000048{\text{AD}}^{2} + 0.002157{\text{AE}}^{2} - 0.000033{\text{BD}}^{2} \\ & - 0.000993{\text{BE}}^{2} + 0.000222{\text{CD}}^{2} - 0.003108{\text{CE}}^{2} \\ \end{aligned}$$

### Crust and crumb colour

**Fig. 3:**
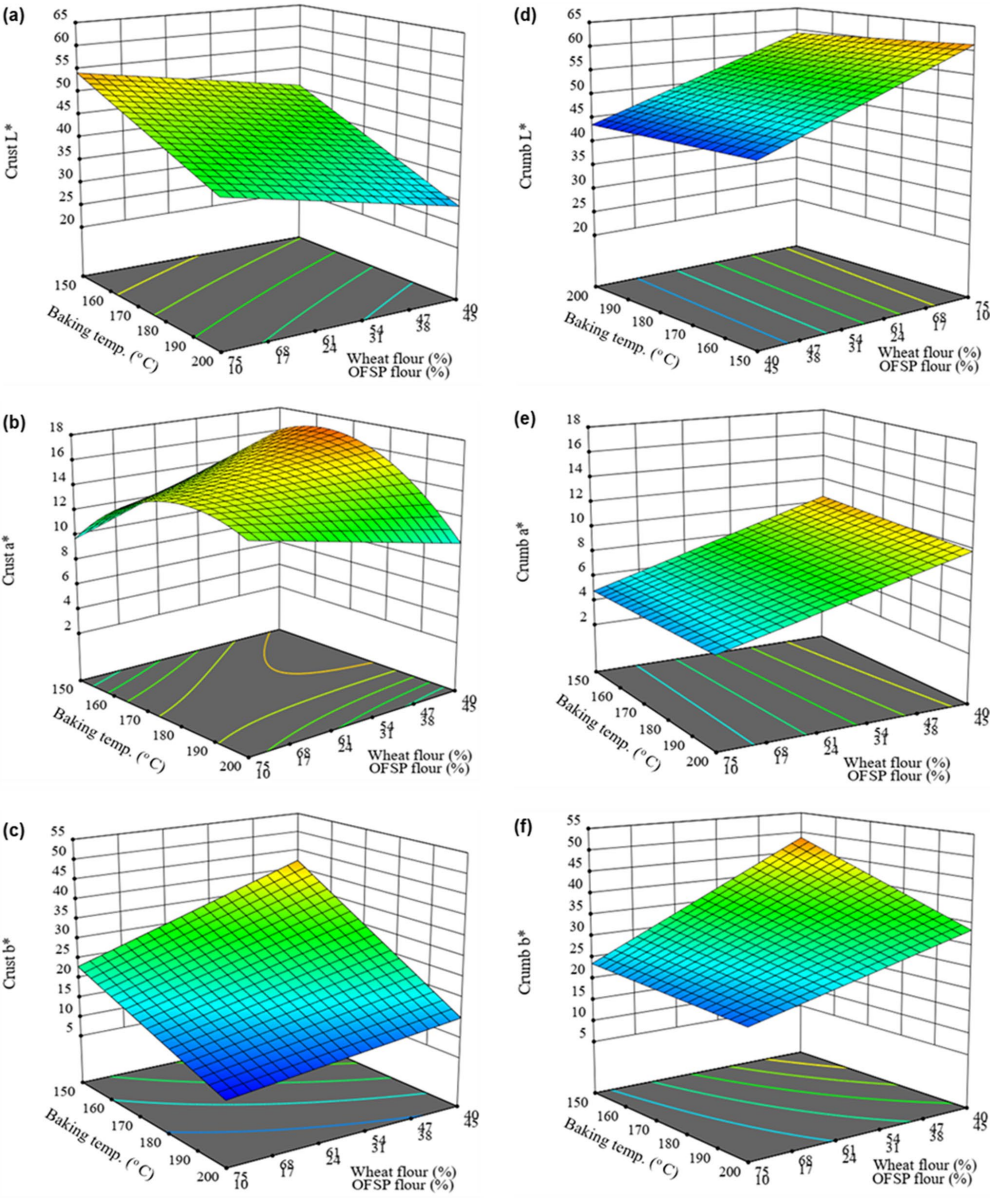
Response surface plots showing the effects of wheat and OFSP flour proportions, pumpkin flour (15%), baking temperature and baking time (19 min) on L^*^, a^*^, and b.^*^ colour values for the composite bread crust (**a**, **b** and **c** respectively) and crumb (**d**, **e** and **f** respectively)

The surface and internal colour of bread are essential quality parameters of bread due to their influence on consumer preference for the product. The bread crust and crumb L* (lightness), a*(redness) and b*(yellowness) values differed widely (p < 0.001) among the various formulations (Table 4). The crust L*, a* and b* values for the control bread were 59.87 ± 0.24, 10.03 ± 0.08, and 4.76 ± 0.12, respectively whereas the crust colour values of the wheat, OFSP and pumpkin composite bread ranged from 23.06 to 59.83 for L*, 7.19 to 17.98 for a* and 8.42 to 49.61 for b*. Mostly, the substitution of wheat flour with OFSP and pumpkin flour caused surface darkening of the composite bread as shown by the declining L* values whereas b* increased (Fig. 3a and c). This could be attributed to the natural pigments such as carotenoids present in OFSP [12] and pumpkin [64]. Values of L* and b* of the crust decreased progressively as the baking conditions increased (Fig. 3a and c). At lower baking temperatures (150–180 °C), the value of a* of the bread crust increased with increasing proportions of OFSP and pumpkin flour whereas a* value declined at higher baking temperatures (≥ 190 °C) with an increasing proportion of the non-wheat flour (Fig. 3b). The changes in crust colour values of the composite bread under different baking conditions can be ascribed to carotenoids degradation [64] and temperature-dependent chemical reactions such as the Maillard reaction [29, 65] and caramelization of sugar [65].

**Table 4:** CIELAB L*, a*, b* colour parameters of the wheat-OFSP-pumpkin composite bread as influenced by flour proportions and baking conditions

Experimental runs	Wheat flour (%)	OFSP flour (%)	Pumpkin flour (%)	Baking temp. (^o^ C)	Baking time (min)	Crust colour			Crumb colour		
						L*	a*	b*	L*	a*	b*
F1	52.5	22.5	25.0	170	19	38.21 ± 0.19^m^	16.57 ± 0.06^d^	22.89 ± 0.15^i^	49.67 ± 0.33^n^	8.77 ± 0.03^i^	34.7 ± 0.11^k^
F2	40.0	50.0	10.0	200	17	32.87 ± 0.25^r^	9.88 ± 0.03^pq^	19.52 ± 0.18^l^	43.30 ± 0.21^v^	9.54 ± 0.05^h^	35.28 ± 0.16^j^
F3	61.9	19.8	18.3	190	23	33.63 ± 0.20^q^	13.51 ± 0.09^k^	11.69 ± 0.21^s^	53.08 ± 0.47^k^	6.63 ± 0.02^l^	24.89 ± 0.09^s^
F4	54.6	33.2	12.2	150	19	50.71 ± 0.15^d^	13.17 ± 0.04^l^	35.46 ± 0.30^c^	51.57 ± 0.61^l^	8.01 ± 0.07^j^	38.29 ± 0.20^e^
F5	80.0	10.0	10.0	180	17	49.80 ± 0.38^f^	14.93 ± 0.02^h^	13.18 ± 0.09^r^	62.25 ± 0.95^c^	4.13 ± 0.02^p^	20.57 ± 0.08^u^
F6	40.0	20.0	40.0	150	17	37.10 ± 0.27^n^	16.32 ± 0.03^e^	35.49 ± 0.17^c^	43.10 ± 0.68^w^	11.87 ± 0.03^a^	49.01 ± 0.19^b^
F7	55.2	10.0	34.8	180	23	27.23 ± 0.24^u^	14.73 ± 0.03^i^	11.35 ± 0.06^t^	50.44 ± 0.13^m^	7.96 ± 0.08^j^	27.60 ± 0.34^q^
F8	40.0	50.0	10.0	150	25	42.98 ± 0.12^j^	15.80 ± 0.05^f^	35.63 ± 0.19^b^	45.02 ± 0.75^q^	10.50 ± 0.03^e^	44.21 ± 0.47^c^
F9	40.0	37.9	22.1	200	15	30.63 ± 0.33^s^	12.01 ± 0.07^n^	17.80 ± 0.05^n^	43.90 ± 0.82^t^	10.72 ± 0.04^d^	36.85 ± 0.19^h^
F10	80.0	10.0	10.0	200	15	44.92 ± 0.18^i^	15.13 ± 0.02^g^	9.27 ± 0.08^u^	61.45 ± 0.29^d^	4.47 ± 0.03^o^	20.50 ± 0.14^u^
F11	80.0	10.0	10.0	200	23	39.45 ± 0.23^l^	15.69 ± 0.08^f^	8.85 ± 0.04^v^	60.58 ± 0.37^e^	3.60 ± 0.02^r^	17.49 ± 0.07^w^
F12	40.0	20.0	40.0	180	21	23.06 ± 0.37^x^	15.87 ± 0.07^f^	14.72 ± 0.13^q^	43.65 ± 0.55^u^	11.05 ± 0.09^c^	35.93 ± 0.09^i^
F13	40.0	50.0	10.0	190	17	36.40 ± 0.14^o^	12.93 ± 0.03^m^	24.90 ± 0.08^g^	43.47 ± 0.31^u^	9.72 ± 0.05^g^	38.24 ± 0.14^e^
F14	43.0	17.0	40.0	170	21	26.79 ± 0.09^v^	18.00 ± 0.12^a^	19.10 ± 0.09^m^	44.38 ± 0.74^s^	10.56 ± 0.08^e^	36.97 ± 0.03^g^
F15	64.2	10.0	25.8	160	21	42.01 ± 0.17^k^	16.85 ± 0.04^c^	20.87 ± 0.10^k^	55.64 ± 0.19^g^	6.61 ± 0.03^lm^	28.05 ± 0.10^p^
F16	52.5	22.5	25.0	170	19	38.19 ± 0.21^m^	16.60 ± 0.03^d^	22.93 ± 0.12^i^	49.64 ± 0.28^n^	8.80 ± 0.05^i^	34.65 ± 0.06^k^
F17	44.8	33.0	22.2	200	19	27.84 ± 0.06^t^	10.64 ± 0.02^o^	11.76 ± 0.08^s^	47.70 ± 0.16^o^	9.67 ± 0.06^g^	31.70 ± 0.06^l^
F18	40.0	50.0	10.0	150	15	51.70 ± 0.14^c^	14.51 ± 0.10^j^	49.61 ± 0.26^a^	44.67 ± 0.24^r^	10.33 ± 0.04^f^	52.86 ± 0.49^a^
F19	59.9	30.1	10.0	160	15	52.82 ± 0.29^b^	13.18 ± 0.08^l^	32.29 ± 0.15^d^	53.90 ± 0.33^i^	7.18 ± 0.09^k^	35.19 ± 0.20^j^
F20	80.0	10.0	10.0	170	21	50.31 ± 0.08^e^	13.50 ± 0.05^k^	16.66 ± 0.03^o^	62.41 ± 0.41^c^	3.98 ± 0.02^q^	18.83 ± 0.04^v^
F21	40.0	50.0	10.0	200	25	24.90 ± 0.10^w^	9.75 ± 0.07^q^	8.42 ± 0.19^w^	43.18 ± 0.08^w^	9.67 ± 0.07^g^	29.42 ± 0.07^n^
F22	70.5	19.5	10.0	190	15	45.70 ± 0.24^g^	14.89 ± 0.02^h^	16.29 ± 0.04^p^	57.60 ± 0.79^f^	5.53 ± 0.02^n^	25.31 ± 0.03^r^
F23	59.9	30.1	10.0	160	25	45.08 ± 0.09^h^	17.01 ± 0.05^bc^	25.17 ± 0.16^f^	53.49 ± 0.56^j^	6.71 ± 0.03^l^	30.24 ± 0.12^m^
F24	45.9	14.1	40.0	160	17	35.62 ± 0.17^p^	17.16 ± 0.09^b^	27.09 ± 0.07^e^	46.76 ± 0.48^p^	11.02 ± 0.02^c^	41.65 ± 0.37^d^
F25	64.2	10.0	25.8	150	21	45.03 ± 0.40^hi^	15.72 ± 0.14^f^	24.53 ± 0.12^h^	55.11 ± 0.32^h^	6.49 ± 0.05^m^	29.23 ± 0.11^o^
F26	80.0	10.0	10.0	150	15	59.83 ± 0.31^a^	7.19 ± 0.07^r^	21.92 ± 0.06^j^	64.19 ± 0.17^b^	4.13 ± 0.04^p^	22.01 ± 0.05^t^
F27	49.2	10.8	40.0	180	15	32.90 ± 0.16^r^	16.92 ± 0.05^c^	17.83 ± 0.18^n^	50.38 ± 0.25^m^	11.17 ± 0.08^b^	37.40 ± 0.21^f^
Control	100.0	0.0	0.0	200	21	59.87 ± 0.24^a^	10.03 ± 0.08^p^	4.76 ± 0.12^x^	74.93 ± 0.38^a^	-0.89 ± 0.02^s^	11.51 ± 0.17^x^

Cross model (flour mixture × baking conditions)	Linear × linear	Linear × quadr	Linear × linear	Linear × linear	Linear × linear	Linear × linear
Model (F-value)	7328.81^***^	5216.07^***^	2023.56^***^	766.08^***^	3188.36^***^	2028.39^***^
Lack-of-fit (F-value)	54.130^ ns^	2.483^ ns^	14.661^ ns^	6.205^ ns^	8.950^ ns^	0.178^ ns^
Coefficient of determination (R^2)^	0.9993	0.9887	0.9989	0.9971	0.9905	0.9981
Adjusted R^2^	0.9975	0.9882	0.9967	0.9958	0.9893	0.9976

Colour values are average ± standard deviation (n = 5). Values within the same column that have different letters are significantly different (p < 0.05). ^***^ P < 0.0001; ns means not significant (p > 0.05); L* lightness; a* redness and b* yellowness

The L*, a* and b* colour values of the bread crumbs differed significantly (p < 0.001) among the different formulations (Table 4). Increasing substitution of wheat flour with OFSP and pumpkin cause a reduction in crumb L* while a* and b* values increased (Fig. 3d, e and f). In other studies, the replacement of wheat flour with potato flour [18, 57], wheat bran dietary fibre [28] increased a* and b* but decrease the L* value of bread crumbs whereas Bultum et al. [38] observed a reduction in values of L* and b* and increased a* when wheat flour was substituted with full-fat rice bran. Although the baking temperature and time affected the crumb colour of the experimental wheat-OFSP-pumpkin composite bread, the effect on the crumb colour was less as compared to the crust.

The experimental data for the L* and b* of the composite bread crust were adequately explained by a linear × linear model whereas the crust a* was sufficiently predicted by a linear × quadratic crossed model. The model fitness statistics are presented in Table 4. The model equations for predicting the crust L*, a* and b values in terms of the proportions of the wheat (A), OFSP (B) and pumpkin flours (C), baking temperature (D) and time (E) are summarized in Eqs. (9–11), respectively.

9 $${\text{Crust L}}^{*} = 1.19081{\text{A}} + 1.25626{\text{B}} + 0.78442{\text{C}} - 0.00301{\text{AD}} - 0.00373{\text{AE}} - 0.00408{\text{BD}} - 0.01101{\text{BE}} - 0.00246{\text{CD}} - 0.02294{\text{CE}}$$

10 $$\begin{aligned} {\text{Crust~a}}^{{\text{*}}} {\text{~}} = & - 1.0832291{\text{A}} - 0.5674866{\text{B}} - 6.7417399{\text{C}} + 0.0175947{\text{AD}} \\ & - 0.0636256{\text{AE}} + 0.013362{\text{BD}} - 0.0165422{\text{BE}} + 0.0394286{\text{CD}} + 0.4317795{\text{CE}} \\ & + 0.0000527{\text{ADE}} + {\text{~}}0.0002623{\text{BDE}} - 0.0017728{\text{CDE}} - 0.0000457{\text{AD}}^{2} \\ & + 0.0015512{\text{AE}}^{2} - {\text{~~}}0.0000612{\text{BD}}^{2} - 0.0007863{\text{BE}}^{2} - 0.0000332{\text{CD}}^{2} - 0.0033625{\text{CE}}^{2} \\ \end{aligned}$$

11 $${\text{Crust b}}^{*} = 0.21465{\text{A}} + 2.49013{\text{B}} + 1.93058{\text{C}} - 0.00123{\text{AD}} + 0.00616{\text{AE}} - 0.00835{\text{BD}} - 0.02851{\text{BE}} - 0.00795{\text{CD}} - 0.02108{\text{CE}}$$

### Crumb textural properties

**Fig. 4:**
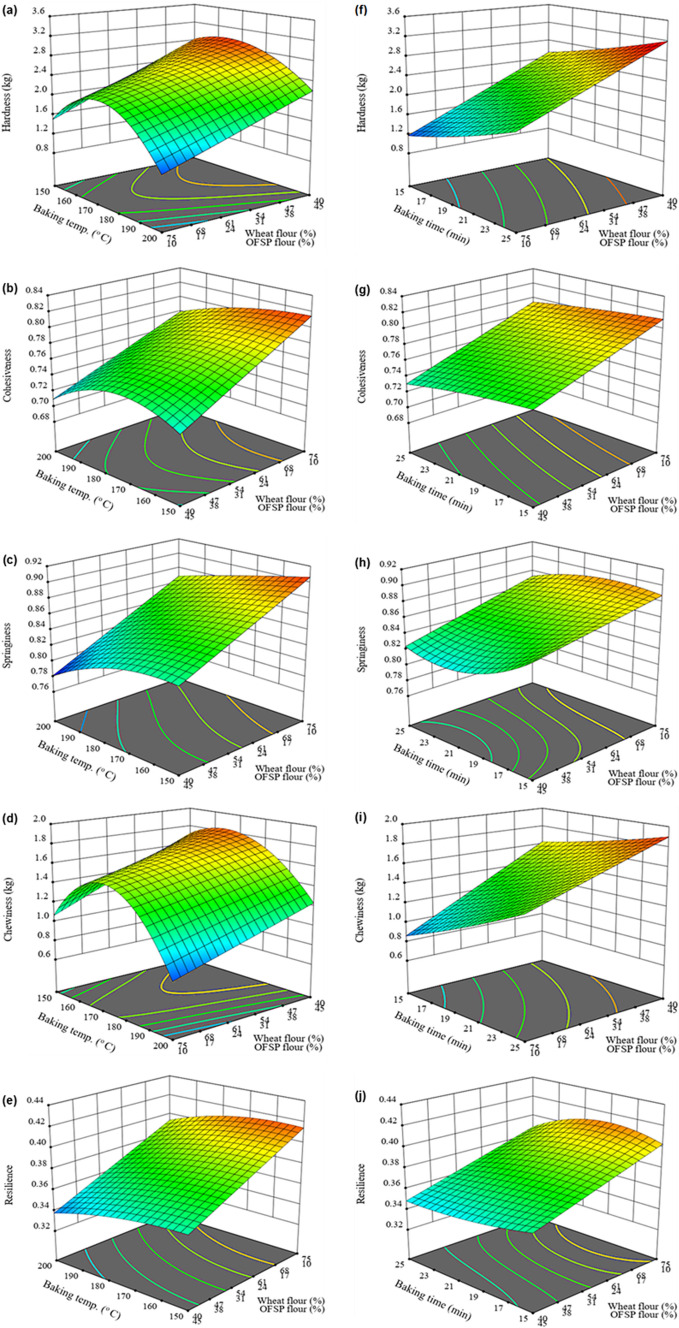
Response surface plots showing the influence of wheat and OFSP flour proportion, pumpkin flour (15%), and baking temperature on hardness (**a**), cohesiveness (**b**) springiness (**c**), chewiness (**d**) and resilience (**e**) as well as the effect of baking time on crumb hardness (**f**), cohesiveness (**g**), springiness (**h**), chewiness (**i**) and resilience (**j**) of the composite bread

Textural characteristics are vital quality attributes used to judge the freshness of bread and influence consumer acceptability [66]. The textural properties of the control and composite bread crumbs are shown in Table 5. The flour mixture and baking conditions had a significant (p < 0.05) on the textural attributes of composite bread crumbs. The values of textural attributes of the composite bread crumbs varied from 1.001 to 3.082 kg for hardness, 0.688 to 0.823 for cohesiveness, 0.781 to 0.913 for springiness, 0.707 to 1.956 for chewiness and 0.326 to 0.424 for resilience. In general, the control bread crumb was more cohesive (0.831 ± 0.003) and springier (0.967 ± 0.001) than the composite bread. Nonetheless, the harness and chewiness values observed in the composite bread were much higher than control (Table 5). The influence of the flour proportions, baking temperature and baking duration as illustrated by response surface plots (Fig. 4). It was noticeable that increasing substitution of wheat flour with OFSP and pumpkin flour increased crumb hardness and chewiness but decreased the cohesiveness, springiness and resilience of the composite bread crumbs (Fig. 4). This phenomenon could be attributed to the dilution of gluten which decreases the formation of gluten network and gas cells and subsequently reduces the amount of gas entrapped in the crumb matrix, minimizes the creation of soft cavity structure within the crumb and increases hardness and decreases springiness of the crumb [28]. Also, the high fibre in the OFSP and pumpkin flour could disrupt starch-gluten interactions resulting in a decrease in the cohesiveness of the crumb [28]. The results of the study are in agreement with the findings by Li et al. [47], who observed a reduction in springiness, cohesiveness and resilience and increased hardness and chewiness of steamed bread enriched with wheat bran. Similarly, the substitution of wheat flour with potato flour increased crumb hardness and chewiness while resilience decreased [57].

**Table 5:** Textural properties of wheat-OFSP-pumpkin composite bread crumbs as affected by flour proportions and baking conditions

Experimental runs	Wheat flour (%)	OFSP flour (%)	Pumpkin flour (%)	Baking temp. (^o^ C)	Baking time (min)	Hardness (kg)	Cohesiveness	Springiness	Chewiness (kg)	Resilience
F1	52.5	22.5	25.0	170	19	2.482 ± 0.002^ h^	0.772 ± 0.001^e^	0.837 ± 0.002^ h^	1.604 ± 0.003^ g^	0.376 ± 0.002^gh^
F2	40.0	50.0	10.0	200	17	2.206 ± 0.002^o^	0.718 ± 0.001^ l^	0.794 ± 0.001^ k^	1.258 ± 0.001^q^	0.349 ± 0.002^ mn^
F3	61.9	19.8	18.3	190	23	2.160 ± 0.001^p^	0.766 ± 0.001^efg^	0.848 ± 0.001^ g^	1.403 ± 0.002^ m^	0.373 ± 0.002^ghi^
F4	54.6	33.2	12.2	150	19	2.285 ± 0.003^ l^	0.761 ± 0.003^ fg^	0.865 ± 0.001^f^	1.504 ± 0.003^j^	0.379 ± 0.002^ fg^
F5	80.0	10.0	10.0	180	17	1.960 ± 0.001^t^	0.816 ± 0.001^ab^	0.895 ± 0.001^c^	1.431 ± 0.002^ l^	0.415 ± 0.002^c^
F6	40.0	20.0	40.0	150	17	2.229 ± 0.001^ m^	0.760 ± 0.001^gh^	0.824 ± 0.001^i^	1.396 ± 0.002^n^	0.347 ± 0.002^ mn^
F7	55.2	10.0	34.8	180	23	3.082 ± 0.004^a^	0.772 ± 0.002^e^	0.822 ± 0.002^ij^	1.956 ± 0.001^a^	0.376 ± 0.001^gh^
F8	40.0	50.0	10.0	150	25	2.783 ± 0.003^c^	0.707 ± 0.004^ m^	0.829 ± 0.001^hi^	1.631 ± 0.004^d^	0.358 ± 0.002^kl^
F9	40.0	37.9	22.1	200	15	2.155 ± 0.003^p^	0.703 ± 0.003^ m^	0.814 ± 0.001^j^	1.233 ± 0.002^r^	0.344 ± 0.003^no^
F10	80.0	10.0	10.0	200	15	1.053 ± 0.001^x^	0.809 ± 0.002^b^	0.891 ± 0.002^ cd^	0.759 ± 0.003^u^	0.401 ± 0.001^d^
F11	80.0	10.0	10.0	200	23	1.076 ± 0.002^w^	0.774 ± 0.001^e^	0.889 ± 0.003^ cd^	0.740 ± 0.001^w^	0.388 ± 0.001^e^
F12	40.0	20.0	40.0	180	21	2.730 ± 0.001^d^	0.746 ± 0.001^ij^	0.789 ± 0.002^kl^	1.607 ± 0.003^ g^	0.341 ± 0.001^no^
F13	40.0	50.0	10.0	190	17	2.607 ± 0.001^f^	0.739 ± 0.004^jk^	0.815 ± 0.002^j^	1.570 ± 0.001^ h^	0.354 ± 0.002^ lm^
F14	43.0	17.0	40.0	170	21	2.981 ± 0.001^b^	0.752 ± 0.003^hi^	0.796 ± 0.001^ k^	1.784 ± 0.001^b^	0.363 ± 0.004^jkl^
F15	64.2	10.0	25.8	160	21	2.383 ± 0.003^i^	0.790 ± 0.001^d^	0.859 ± 0.002^f^	1.617 ± 0.002^f^	0.387 ± 0.001^ef^
F16	52.5	22.5	25.0	170	19	2.483 ± 0.002^ h^	0.771 ± 0.001^e^	0.837 ± 0.002^ h^	1.602 ± 0.003^ g^	0.374 ± 0.002^gh^
F17	44.8	33.0	22.2	200	19	1.847 ± 0.003^u^	0.720 ± 0.003^ l^	0.789 ± 0.001^kl^	1.049 ± 0.001^t^	0.338 ± 0.001^o^
F18	40.0	50.0	10.0	150	15	2.555 ± 0.002^ g^	0.734 ± 0.001^ k^	0.866 ± 0.001^ef^	1.624 ± 0.003^e^	0.367 ± 0.003^ij^
F19	59.9	30.1	10.0	160	15	2.071 ± 0.001^r^	0.784 ± 0.001^d^	0.883 ± 0.003^d^	1.434 ± 0.002^ l^	0.385 ± 0.002^ef^
F20	80.0	10.0	10.0	170	21	2.093 ± 0.003^q^	0.815 ± 0.001^ab^	0.913 ± 0.004^b^	1.557 ± 0.002^i^	0.424 ± 0.002^b^
F21	40.0	50.0	10.0	200	25	2.218 ± 0.003^n^	0.688 ± 0.001^n^	0.781 ± 0.001^ l^	1.192 ± 0.001^ s^	0.326 ± 0.002^p^
F22	70.5	19.5	10.0	190	15	1.826 ± 0.001^v^	0.800 ± 0.002^c^	0.874 ± 0.001^e^	1.277 ± 0.001^p^	0.390 ± 0.002^e^
F23	59.9	30.1	10.0	160	25	2.353 ± 0.001^ k^	0.768 ± 0.001^efg^	0.860 ± 0.003^f^	1.554 ± 0.001^i^	0.373 ± 0.004^ghi^
F24	45.9	14.1	40.0	160	17	2.362 ± 0.001^j^	0.769 ± 0.001^ef^	0.821 ± 0.002^ij^	1.491 ± 0.004^ k^	0.365 ± 0.001^ijk^
F25	64.2	10.0	25.8	150	21	1.973 ± 0.001^ s^	0.791 ± 0.001^d^	0.866 ± 0.002^ef^	1.352 ± 0.001^o^	0.393 ± 0.002^de^
F26	80.0	10.0	10.0	150	15	1.001 ± 0.001^y^	0.823 ± 0.001^a^	0.907 ± 0.002^b^	0.747 ± 0.003^v^	0.412 ± 0.003^c^
F27	49.2	10.8	40.0	180	15	2.648 ± 0.002^e^	0.752 ± 0.001^hi^	0.828 ± 0.002^i^	1.649 ± 0.002^c^	0.370 ± 0.004^hij^
control	100.0	0.0	0.0	200	21	0.573 ± 0.004^z^	0.831 ± 0.003^a^	0.967 ± 0.001^a^	0.460 ± 0.003^x^	0.449 ± 0.002^a^
P-value						0.0000	0.000	0.0000	0.0000	0.000

Values indicate average ± standard deviation (n = 3). Values within the same column that have different letters are significantly different (p < 0.05)

The baking temperature and time have a significant (p < 0.05) influence on the textural properties of the composite bread (Fig. 4a-e and f-j). Mostly, an increase in baking temperature from 150 to 170 °C caused an increase in the hardness and chewiness of the crumb whereas higher baking temperatures (> 170 °C) decreased the hardness and chewiness of the final baked bread (Fig. 4a and d). This observation could be attributed to the decrease in crumb moisture as the baking temperature increased from 150 to 180 °C and the subsequent increase in crumb moisture at higher baking temperatures (≥ 180 °C) as shown in Fig. 2c. Moreover, decreasing trends for crumb cohesiveness, springiness and resilience were observed with increased baking temperature (Fig. 4b, c and e). Also, increasing the baking time led to a significant increase in crumb hardness and chewiness (Fig. 4i and f) and a decrease in cohesiveness, springiness and resilience of the composite bread (Fig. 4g, h and j). This could be ascribed to the increased loss of moisture from the crumb due to extended exposure to heat. The results of the current study were in agreement with the findings by Shen et al. [67], who reported that higher baking temperatures decreased crumb hardness whereas prolonged baking increased crumb hardness. Similarly, an increase in crumb firmness due to lengthy baking time was reported in a previous study [29]. The textural attributes of the formulated composite bread crumbs were adequately explained by a linear × quadratic cross model summarized in Eq. (12).

12 $$\begin{gathered} {\text{Y}} = \, {\upbeta }_{1} {\text{A}} + {\upbeta }_{2} {\text{B}} + {\upbeta }_{3} {\text{C}} + {\upbeta }_{4} {\text{AD}} + {\upbeta }_{5} {\text{AE}} + {\upbeta }_{6} {\text{BD}} + {\upbeta }_{7} {\text{BE}} + {\upbeta }_{8} {\text{CD}} + {\upbeta }_{9} {\text{CE}} + {\upbeta }_{10} {\text{ADE}} \hfill \\ + \, {\upbeta }_{11} {\text{BDE}} + {\upbeta }_{12} {\text{CDE}} + {\upbeta }_{13} {\text{AD}}^{2} + {\upbeta }_{14} {\text{AE}}^{2} + {\upbeta }_{15} {\text{BD}}^{2} + {\upbeta }_{16} {\text{BE}}^{2} + {\upbeta }_{17} {\text{CD}}^{2} + {\upbeta }_{18} {\text{CE}}^{2} \hfill \\ \end{gathered}$$

where Y is the textural parameter, A, B, and C are levels of wheat, OFSP and pumpkin flour in the flour mixture respectively, D and E are baking temperature and time respectively, β_1_, β_2,_ β_3_…………..β18 represent the regression coefficients of the model terms. Table 6 shows the regression coefficients of the model terms and fitness statistics for the prediction of hardness, cohesiveness, springiness, chewiness and resilience values of bread products as influenced by the processing factors in terms of the actual setting. The developed models and a large number of the model terms are significant (p < 0.05). The model fitness statistics for all the textural parameters showed higher values for R^2^ and adjusted R^2^ (0.9812–0.9987), prediction R^2^ (0.8813–9695), adequate precision (29.20–94.61) and lower coefficient of variance (0.23–2.07). The higher R^2^ and adequate precision (> 4) suggest the developed models have a higher degree of accuracy [52]. Therefore, the established models could be useful for suitable prediction of the textural attributes of wheat, OFSP and pumpkin flour composite bread as affected by the flour blending ratio, baking temperature and baking time.

**Table 6:** Regression coefficients of crossed-model (Linear × quadratic) and fitness statistics for predicting the textural properties of the bread crumbs in terms of actual settings of processing factors

	Hardness	Cohesiveness	Springiness	Chewiness	Resilience
Model terms					
A	−0.5876001^***^	0.00294835^***^	0.01215257^***^	−0.4499320^***^	−0.00224923^***^
B	0.1291523^***^	−0.02265566^***^	−0.00505028^***^	0.0530513^***^	0.00950264^***^
C	0.0409827^***^	0.03066094^***^	0.03145016^***^	0.1884075^***^	−0.00916456^***^
AD	0.0057341^*^	0.00002520^***^	−0.00011884^**^	0.0043653^**^	0.00002155^*^
AE	0.0106324^ ns^	0.00043800^***^	0.00082971^ ns^	0.0086415^ ns^	0.00053966^*^
BD	−0.0017581^***^	0.00032620^***^	0.00030078^***^	−0.0006618^***^	−0.00007589^**^
BE	0.0077952^*^	0.00011974^***^	−0.00117132^***^	0.0041027^ ns^	0.00006135^**^
CD	0.0046925^***^	-0.00006605^*^	−0.00007375^ ns^	0.0016114^**^	0.00029584^*^
CE	−0.043115^***^	−0.00183788^*^	−0.00188239^*^	−0.0321646^***^	−0.00134759^ ns^
ADE	−0.000006^*^	−0.00000237^***^	−0.00000078^ ns^	−0.0000104^**^	−0.00000032^ ns^
BDE	0.0000158^ ns^	−0.00000037^ ns^		0.0000155 ns	−0.00000052^*^
CDE	−0.0000839^*^	0.00000989^***^	0.00000402^*^	−0.0000404^*^	
AD^2^	−0.0000160^***^	0.00000004^*^	0.00000037^*^	−0.0000119^***^	−0.00000005^ ns^
AE^2^	-0.0002538^*^	−0.00000131^ ns^	−0.00001739^*^	-0.0001784^*^	−0.00001278^*^
BD^2^	0.0000038^**^	−0.00000090^***^	−0.00000091^*^	0.0000007^**^	0.00000024^ ns^
BE^2^	−0.0002722^*^	−0.00000258^ ns^	0.00002763^**^	−0.0001823^*^	
CD^2^	−0.0000101^***^	−0.00000039^**^		−0.0000033^**^	−0.00000089^**^
CE^2^	0.0015845^***^	0.00000427^ ns^	0.00002874^*^	0.0010692^***^	0.00003558^*^
Fitness statistics					
F-value (model)	212.79^***^	650.95^***^	227.29^***^	165.94^***^	59.48^***^
F-value (lack-of-fit)	4.850^ ns^	6.612^ns^	6.504^ns^	9.630^ns^	9.24^ns^
* R*^*2*^	0.9975	0.9987	0.9965	0.9968	0.9878
Adjusted *R*^*2*^	0.9928	0.9974	0.9917	0.9908	0.9812
Prediction R^2^	0.9219	0.9695	0.9669	0.9013	0.8812
Adequate precision	56.61	94.61	48.53	51.82	29.20
C.V %	2.02	0.23	0.42	2.07	1.11

Where A, B, C represent wheat, OFSP and pumpkin flour respectively, D is the baking temperature, E is the baking time, coefficient of determination (R^2^), C.V is the coefficient of variance, *p < 0.05; **p < 0.001; ***p < 0.0001

### Influence of processing factors on bread staling

**Fig. 5:**
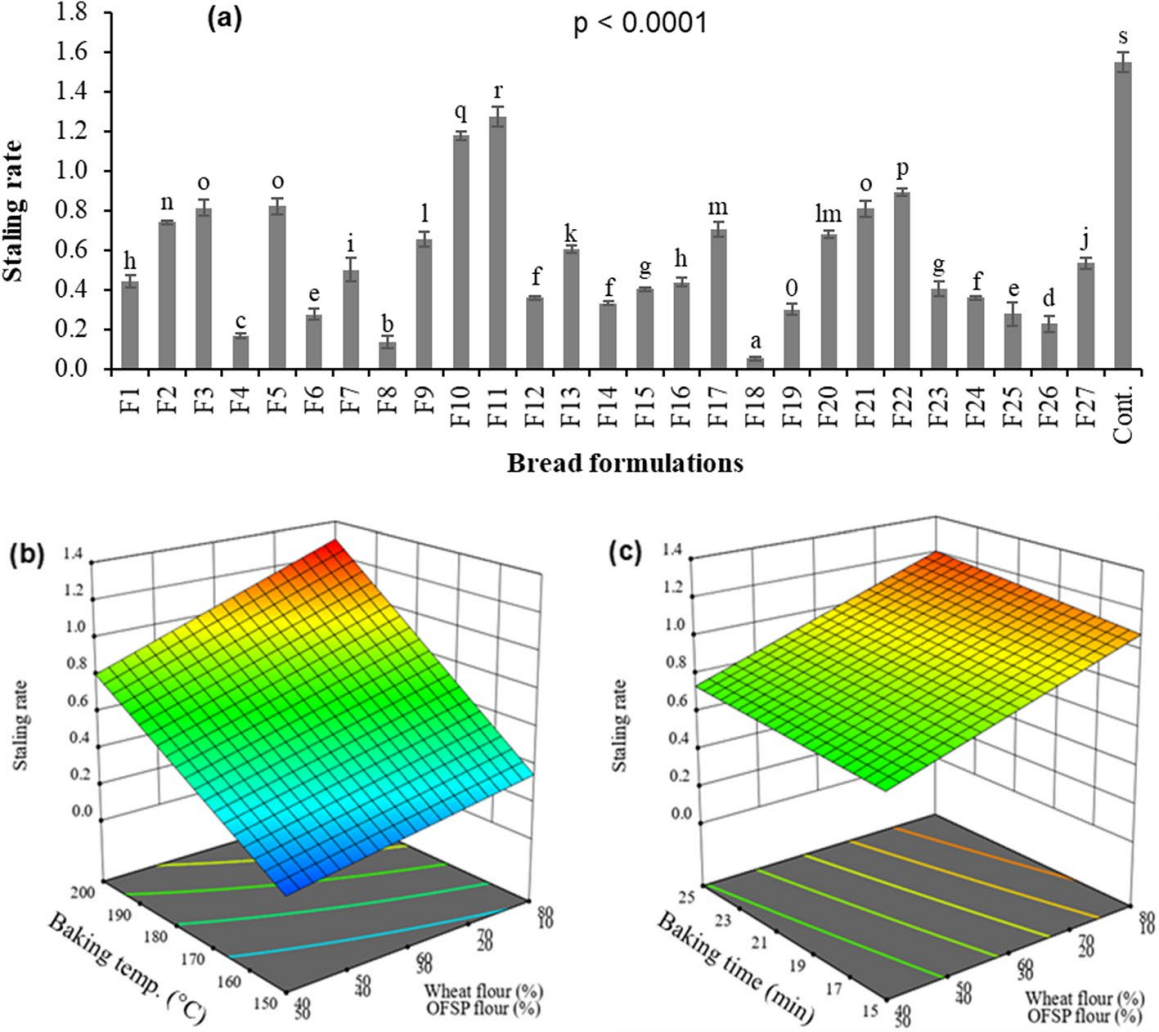
Staling rate on the crumb staling rate after 24 h of storage (**a**) and response surface plots showing the influence of baking temperature (**b**) and baking time (**c**) on the staling rate of composite bread. Data presented are average values of three replicated measurements. Error bars represent standard deviation Values with different lower case letters are significantly different (p < 0.05)

Changes in bread such as crumb firming during storage could influence consumer acceptability of the product [30]. The crumb firming or staling behaviour of the bread samples after 24 h of storage as influenced by flour mixture and baking conditions is shown in Fig. 5. The staling rate in the crumbs varied between 0.10 and 1.28 for composite bread and 1.56 ± 0.054 for the control (Fig. 5a). At a fixed baking temperature and time, the rate of crumb staling declined with increasing addition of OFSP and pumpkin flour in the bread formula (Fig. 5b and c). In general, retrogradation of amylopectin, gluten-starch interaction [31] and migration of moisture from crumb to crust in a crusty [30] are the major causes of staling in bread. Therefore, the decline in staling rate of bread crumbs with increasing proportions of non-conventional flour could be due to higher dietary fibre and the lower starch retrogradation capacity of OFSP flour [12], and pumpkin starch [22, 68]. Besides, the response surface plots showed a linear relationship between bread staling rate and baking conditions (Fig. 5b and c). The higher the baking temperature and time, the greater the crumb staling rate during storage. Mostly, baking at higher temperatures for a longer period produces thicker and dried bread crust which facilitates moisture movement from the crumb to the crust during storage and increases the crumb's firmness [30].

### Relationship between quality characteristics of dough and bread

**Fig. 6:**
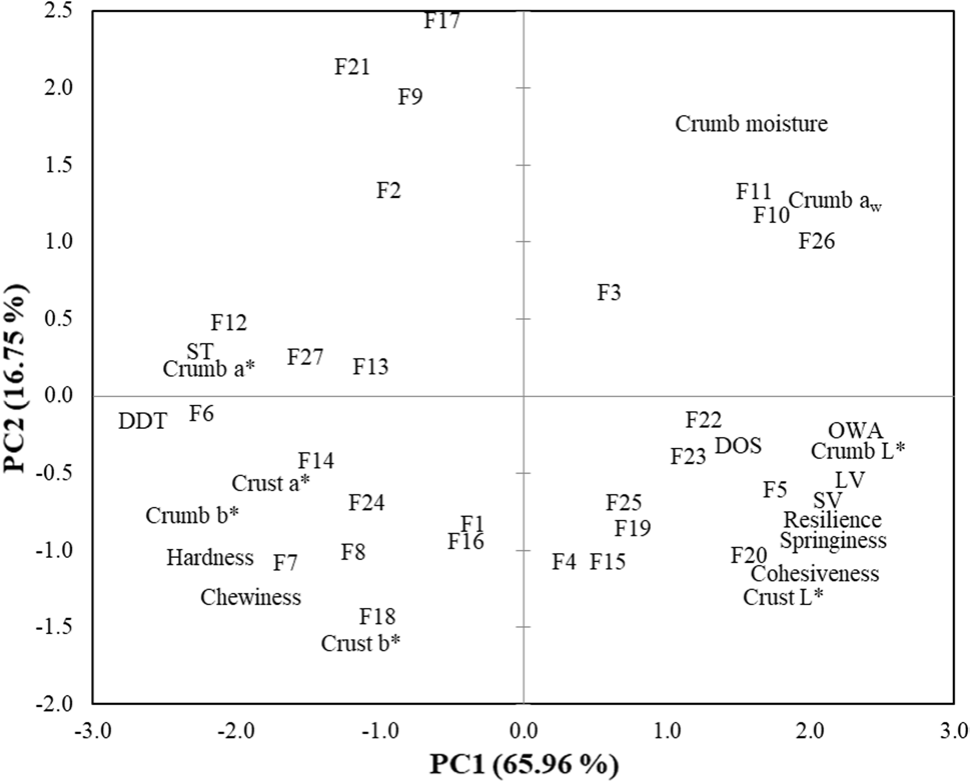
Biplot of the principal component analysis of the dough and bread quality characteristics of the various wheat, OFSP and pumpkin composite bread formulations. *a*_*w*_ water activity, *DDT* dough development time, *DOS* dough degree of softening, *OWA* optimum water absorption, *ST* stability time, *LV* loaf volume, *SV* specific volume. F1, F2, F3, F4, F5, F6, F7, F8, F9, F10,…..F27 indicates the bread formulations as shown in Table 1

A biplot of the principal component analysis (PCA) of the wheat, OFSP and pumpkin composite dough and bread quality properties is shown in Fig. 6. The PCA has revealed that two principal components defined 82.71% of the variation in the original data whereby 65.96% and 16.75% of the variance were explained by the first component (PC1) and a second component (PC2), respectively. The positive axis of PC1 (x-axis) was typically described by OWA, loaf volume, specific volume, crust L*, crumb L*, cohesiveness, springiness and resilience. These quality properties were dominant among samples F5 and F20 (Fig. 6). The F5 and F20 bread samples which were produced from the same flour mixtures but different baking conditions had similar quality characteristics due to their close association in the PCA biplot. This observation was in agreement with previous findings that different baking conditions could produce bread with similar physical properties [29]. Generally, the negative axis of PC1 was defined by ST, DDT, a*, b*, hardness and chewiness. Mostly, DDT and crust a* are dominant characteristics in formulations F6 and F12 whereas crumb hardness and chewiness were major quality attribute found in sample F7. Moreover, the PC2 (y-axis) of the PCA biplot was best described by crumb moisture and a_w_ on the positive axis and these attributes of the bread were prominent in samples F10, F11 and F26. The wide distribution of the samples and their quality attributes in the four quadrants of the PCA biplot revealed the variations in the formulated bread products in terms of the investigated quality attributes.

Additionally, the relationship between the dough and bread quality properties was established by the PCA. The OWA had a significant (p < 0.01) positive correlation with crumb moisture content (r = 0.689) and a_w_ (r = 0.776). Furthermore, OWA, loaf volume, specific volume, crumb cohesiveness, springiness and resilience have strong positive relationships (r = 0.755–0.945, p < 0.01) whereas crumb hardness and chewiness also exhibited a very strong positive correlation (r = 0.944, p < 0.01). Nonetheless, OWA, specific volume, and springiness showed a strong negative correlation with crumb hardness (r = −0.764 to −0.937, p < 0.01). This study has found that some properties of dough namely OWA could be applied to predict the physical and textural properties of the composite bread.

### Optimization of the processing factors

The optimization goal was to maximize dough water absorption, loaf volume, specific volume, crumb cohesiveness, springiness, and resilience, and to minimize hardness, chewiness and staling. The optimization also aimed at achieving target values for crust colour (L*, a*, b*), and crumb moisture. A crust L* of 50 was targeted based on the acceptable top crust L* value for white-sandwich bread [69] whereas the mid-range values were used as target values for crust a*, b* and crumb moisture. Moreover, due to the high negative effects of extremely high baking temperatures and longer baking times on the quality characteristics of the composite bread, the goal was to minimize baking temperature while baking time was maintained within the design values since reducing both processing conditions can have detrimental effects on the product as well. The optimum formula for the composite bread was 78.5% wheat flour, 11.5% OFSP flour, 10.0% pumpkin flour, a baking temperature of 160 °C and a baking time of 20 min (Table 7). This optimization solution had a global desirability value of 0.76. The optimal processing factors and a comparison between the quality attributes of the optimal wheat-OFSP-pumpkin composite bread formulation and the control bread samples are presented in Table 7. From the results, the optimized composite flour dough had slightly lower OWA and higher DDT values compared with the control. Moreover, the control bread sample showed higher values for a specific volume, L*, crumb a_w_, cohesiveness, springiness, chewiness and resilience but recorded lower values for a*, b* and hardness as compared with the optimized wheat-OFSP-pumpkin composite bread sample (Table 7).

**Table 7:** Optimization criteria and predicted values of the dough and bread quality properties of the optimized composite bread formula

Factor/Response	Optimization goal	Lower level	Upper level	Target value	Importance	Desirability value (*d*_*i*_)	Optimal value	Control bread sample
Factor/Response								
Wheat flour (%)	Keep in range	40	80		–	1.0	78.5	100
OFSP flour (%)	Keep in range	10	50		–	1.0	11.5	0.0
Pumpkin flour (%)	Keep in range	10	40		–	1.0	10.0	0.0
Baking temp. (^o^C)	Minimize	150	200		5	1.0	160.0	200
Baking time (min)	Keep in range	15	25		–	1.0	20.0	21
OWA (%)	Maximize	50.80	58.21		5	0.99	58.10^a^	60.30^b^
DDT (min)	Minimize	2.60	29.20		5	0.98	3.30^b^	2.10^a^
LV (cm^3^)	Maximize	166.53	349.94		5	0.88	331.50^a^	363.78^b^
SV (cm^3^/g)	Maximize	1.34	2.62		5	0.93	2.54^a^	2.86^b^
Crust L*	Target	23.06	60.71	50.00	5	0.73	53.4^a^	59.87^b^
Crust a*	Target	7.19	17.98	12.59	5	0.99	12.13^b^	10.03^a^
Crust b*	Target	8.42	49.45	28.94	5	0.55	19.85^b^	4.76^a^
Crumb MC (%)	Target	25.20	37.33	31.26	5	0.86	27.23^a^	31.20^a^
Crumb a_w_	Minimize	0.853	0.908		5	0.30	0.890^a^	0.916^b^
Hardness (kg)	Minimize	1.010	3.082		5	0.57	1.936^b^	0.573^a^
Cohesiveness	Maximize	0.688	0.823		5	0.96	0.818^a^	0.831^b^
Springiness	Maximize	0.781	0.913		5	0.98	0.912^a^	0.967^b^
Chewiness (kg)	Minimize	0.707	1.956		5	0.42	1.447^b^	0.460^a^
Resilience	Maximize	0.326	0.424		5	0.96	0.422^a^	0.449^b^
Global desirability (*D*)						0.76		

Dough and bread quality attributes of optimal formulation and control with different superscript letters are significantly different (p < 0.05). OWA is the optimum water absorption, DDT is dough development time, LV is loaf volume, SV is specific volume and MC is moisture content. L*, a* and b* are CIELAB colour attributes for lightness, redness and yellowness respectively

## Conclusion

Dough and bread quality characteristics as influenced by wheat flour substitution with OFSP and pumpkin flours and baking conditions were successfully studied and optimized using response surface methodology. The addition of OFSP and pumpkin flour greatly reduced dough OWA, loaf bread specific volume, crumb a_w_, L*, cohesiveness, springiness, resilience and staling rate whereas dough development time, crumb hardness and chewiness values were augmented. Furthermore, the loaf volume and specific volume of the composite bread deteriorated greatly while crumb moisture increased as the baking temperature exceeded 180 °C. The crust a* value intensified with increasing baking temperature but declined greatly as the baking temperature exceeded 180 °C. The incorporation of OFSP and pumpkin flour in the bread formulation reduce staling rate in the composite bread during storage. Inversely, higher baking temperatures (≥ 190 °C) and longer baking time (≥ 21 min) increased crumb staling in stored bread. The optimized composite bread formulation was 78.5% wheat flour, 11.5% OFSP flour, 10.0% pumpkin flour, and baking conditions of 160 °C for 20 min. The results obtained in this study could be useful in the bakery industry for the production of functional bread products. Nonetheless, some limitations relating to the industrial application of OFSP and pumpkin flour include the commercial availability of OFSP and pumpkin flour, the stability of bioactive compounds such as carotenoids during flour production and storage, as well as the degradation of carotenoids during bread baking and storage processes. Moreover, the industrial application of OFSP and pumpkin flour for bread baking may require the modification of existing bread-making procedures of manufacturers. It is recommended that future research should focus on the assessment of the stability of bioactive compounds of the optimized wheat-OFSP-pumpkin composite bread during baking and storage.

## Data Availability

The datasets generated during the current study are available from the corresponding author on reasonable request.
